# On the Monotonicity of Relative Entropy: A Comparative Study of Petz’s and Uhlmann’s Approaches

**DOI:** 10.3390/e27090954

**Published:** 2025-09-14

**Authors:** Santiago Matheus, Francesco Bottacin, Edoardo Provenzi

**Affiliations:** 1 Dipartimento di Matematica, Università degli Studi di Padova, Via Trieste 63, 35121 Padova, Italy; santiagojavier.matheusrocha@studenti.unipd.it (S.M.); bottacin@math.unipd.it (F.B.); 2 Institute of Mathematics, Université de Bordeaux, CNRS, Bordeaux INP, IMB, UMR 5251, 351 Cours de la Libération, F-33400 Talence, France

**Keywords:** quantum relative entropy, monotonicity, data processing inequality

## Abstract

We revisit the monotonicity of relative entropy under the action of quantum channels, a foundational result in quantum information theory. Among the several available proofs, we focus on those by Petz and Uhlmann, which we reformulate within a unified, finite-dimensional operator-theoretic framework. In the first part, we examine Petz’s strategy, identify a subtle flaw in his original use of Jensen’s contractive operator inequality, and point out how it was corrected to restore the validity of his line of reasoning. In the second part, we develop Uhlmann’s approach, which is based on interpolations of positive sesquilinear forms and applies automatically to non-invertible density operators. By comparing these two approaches, we highlight their complementary strengths: Petz’s method is more direct and clear; Uhlmann’s method is more abstract and general. Our treatment aims to clarify the mathematical structure underlying the monotonicity of relative entropy and to make these proofs more accessible to a broader audience interested in both the foundations and applications of quantum information theory.

## 1. Introduction

The (quantum) relative entropy is a central concept in quantum information theory, quantifying the distinguishability between quantum states and serving as a key tool in the analysis of information processing tasks.

One of its most important properties is the *monotonicity under quantum channels* (a quantum channel is a completely positive trace-preserving linear map; see later for a rigorous definition), which expresses the idea that state distinguishability cannot increase during the dynamical evolution of a quantum system that interacts with an environment. This property is also known as the data processing inequality (DPI).

The concept of relative entropy was first introduced by Umegaki in 1962 [1] in the setting of σ-finite von Neumann algebras, and it was later extended to arbitrary von Neumann algebras by Araki in 1976 [2] by means of Tomita–Takesaki modular theory.

The proof of the monotonicity of relative entropy is closely related to the proof of the strong subadditivity (SSA) of the von Neumann entropy. The latter serves as a measure of the mixedness of quantum states, and the validity of the SSA ensures that quantum uncertainty behaves consistently across composite systems, placing fundamental constraints on how information and correlations can be distributed among subsystems.

The first step toward proving SSA was made in 1968 by Lanford and Robinson [3], who established the subadditivity of the von Neumann entropy and conjectured its stronger form. In 1973, Lieb [4], building on earlier work by Wigner, Yanase, and Dyson, proved several key properties concerning the convexity and concavity of operator functions and trace functionals.

These results enabled Lieb and Ruskai to establish the full proof of the strong subadditivity of the von Neumann entropy for both finite and infinite-dimensional Hilbert spaces later that same year in their landmark paper [5]. In that work, they also derived, though without emphasizing it as such, the monotonicity of relative entropy under the partial trace operation, which constitutes a special case of quantum channels.

The first explicit and general proof of the monotonicity of relative entropy under the action of quantum channels was provided two years later by Lindblad in his seminal 1975 paper [6], thanks to the results established by Lieb and Ruskai in [5].

A further breakthrough was achieved by Uhlmann in 1977, who extended the property of monotonicity to a broader class of transformations: the adjoints of unital Schwarz maps [7].

The equivalence between SSA and the monotonicity of relative entropy was rigorously established by Petz in the 1980s [8,9]. Later, in [10], Petz proposed a new proof of monotonicity and posed the question of whether this property also holds for positive (but not necessarily completely positive) trace-preserving linear maps. This question remained open for several years until it was affirmatively resolved in 2023 by Müller-Hermes and Reeb in [11]. Other pertinent references related to the monotonicity of relative entropy are [12,13,14,15].

In this paper, we focus specifically on the demonstration strategies proposed by Petz and Uhlmann in [10] and [7] respectively, which we believe offer particularly interesting and useful complementary perspectives. Petz’s proof relies on operator-algebraic methods and an explicit representation of the relative entropy in terms of a suitable inner product, an idea inspired by the previously quoted work of Araki.

However, as we show, his original use of Jensen’s operator inequality contains a subtle flaw when applied in its contractive form. We point out how Petz himself and Nielsen corrected the problem in order to restore the validity of Petz’s original approach.

Uhlmann’s proof, on the other hand, is formulated in terms of interpolations of positive sesquilinear forms, a technique that naturally extends to non-invertible states and arbitrary quantum channels.

Despite their foundational importance, both Petz’s and Uhlmann’s proofs are often considered technically demanding and conceptually opaque. Petz’s approach, while elegant, involves intricate manipulations of operator inequalities that can obscure the overall structure of the argument. Uhlmann’s method introduces a formalism that is unfamiliar to many working in quantum information theory and is rarely presented in full detail in the literature.

We aim to clarify and systematize both strategies by reformulating them in a unified, finite-dimensional, operator-theoretic framework, thus making both proofs more accessible to a wider audience.

This paper is structured as follows: In Section 2, we recall the necessary preliminaries on operator convexity, partial trace, and quantum channels. In Section 3, we analyze Petz’s proof, highlight its limitations, and discuss its correction. In Section 4, we develop Uhlmann’s approach in detail and derive the monotonicity of relative entropy in the general setting.

## 2. Mathematical Preliminaries

In this section, we start recalling the basic definitions and results needed for the rest of the paper.

Given a finite-dimensional Hilbert state space $\left(H,{\langle \;,\;\rangle }_{H}\right)$ over the field F=R or C, B(H) indicates the F-algebra of linear (bounded) operators A:H→H.

We recall that A∈B(H) is as follows:

- *Positive semi-definite*, written as A≥0 if ${\langle x,Ax\rangle }_{H}\geq 0$ for all x∈H;
- *Positive definite*, written as A>0 if ${\langle x,Ax\rangle }_{H}>0$ for all $x\neq 0_{H}\in H$;
- *Hermitian* if $A=A^{†}$, where $A^{†}$ is the *adjoint* operator of *A*, defined by the formula ${\langle A^{†}x,y\rangle }_{H}={\langle x,Ay\rangle }_{H}$ for all x,y∈H.

If F=C, then a positive semi-definite, or positive definite, operator is automatically Hermitian, but this is not the case if F=R.

Suppose now that H is the state space of a quantum system. The density operator (also called the density matrix) ρ∈B(H) associated with a given state *s* of the system is positive semi-definite, Hermitian, and such that Tr(ρ)=1. Hence, ρ has eigenvalues $0\leq \lambda_{j}\leq 1$, which sum up to 1.

As is well-known, if *s* is a pure state, then ρ is a rank-one orthogonal projector and thus not invertible.

B(H) itself becomes an F-Hilbert space when it is endowed with the Hilbert–Schmidt operator inner product

(1) $${\langle A,B\rangle }_{B(H)}:=\mathrm{Tr}\left(A^{†}B\right),\;A,B\in B\left(H\right).$$

The subset of B(H) given by Hermitian operators on H, indicated with $B_{H}\left(H\right)$, is a real Hilbert space with respect to the inner product inherited from B(H), and it is also a partially ordered set with respect to the *Löwner ordering*, defined as follows: for all $A,B\in B_{H}\left(H\right)$, A≤B⇔B−A≥0.

Given a function f:I⊆R→R and $A\in B_{H}\left(H\right)$ with spectral decomposition $A=UDU^{†}$, with *U* being unitary and *D* being diagonal, with entries given by the eigenvalues $\lambda_{j}$ of *A*, all supposed to belong to *I*, we write as usual

(2) $$f\left(A\right)=Uf\left(D\right)U^{†},$$

where f(D) is diagonal, with non-trivial entries given by $f(\lambda_{j})$.

If, for every finite-dimensional H and every couple of operators $A,B\in B_{H}\left(H\right)$, we have

(3) A≤B⇒f(A)≤f(B),

then *f* is said to be *operator monotone* on *I*. Instead, if we have

(4) $$f\left(\frac{A+B}{2}\right)\leq \frac{f(A)+f(B)}{2},$$

then *f* is said to be *operator convex* on *I*. If the last inequality is reversed, *f* is said to be *operator concave* on *I*.

By Löwner’s theorem (see, e.g., [16] chapter V.4), f:I→R is operator monotone if and only if it has an analytic continuation that maps the upper half-plane $H_{+}$ into itself. Notable examples of operator monotone functions on (0,+∞) are x↦log(x) and x↦−1/x.

If f:I→R is a continuous and operator monotone function on *I*, then ∀a∈I, the function F:I→R given by

(5) $$F\left(x\right)=\int_{a}^{x}f\left(t\right)dt$$

is operator convex on *I*; see again [16]. So, since t↦−1/t is operator monotone on (0,+∞),

(6) $$x⟼\int_{1}^{x}-\frac{1}{t}dt=-\log\left(x\right)$$

is operator convex on (0,+∞), which implies that x↦log(x) is operator concave on (0,+∞).

As is well-known, a convex function f:I→R satisfies the Jensen inequality

(7) $$f\left(\sum_{i=1}^{n}\lambda_{i}x_{i}\right)\leq \sum_{i=1}^{n}\lambda_{i}f\left(x_{i}\right),$$

for all $x_{1},\ldots ,x_{n}\in I$ and non-negative weights $\lambda_{1},\ldots ,\lambda_{n}$ such that $\sum_{i=1}^{n}\lambda_{i}=1$.

The Jensen inequality can be generalized to operator convex functions, as stated in the following celebrated theorem, proven by Hansen and Pedersen [17].

**Theorem** **1** **(Jensen’s operator inequality).** *For a continuous function f:I→R, the following conditions are equivalent:*

*(i)*  *f is operator convex on I.*
*(ii)*  *For each natural number n≥1, the following inequality holds:*
  (8) $$f\left(\sum_{i=1}^{n}A_{i}^{†}X_{i}A_{i}\right)\leq \sum_{i=1}^{n}A_{i}^{†}f\left(X_{i}\right)A_{i},$$
  *for every n-tuple of bounded Hermitian operators $X_{1},\ldots ,X_{n}$ on an arbitrary Hilbert space H, with spectra contained in I, and every n-tuple of operators $\left(A_{1},\ldots ,A_{n}\right)$ on H satisfying $\sum_{i=1}^{n}A_{i}^{†}A_{i}=id_{H}.$*
*(iii)*  *$f\left(V^{†}XV\right)\leq V^{†}f\left(X\right)V$ for every isometry V:K→H from an arbitrary Hilbert space K on an arbitrary Hilbert space H and every Hermitian operator X on H with spectrum contained in I.*

An immediate consequence of this theorem is the following corollary, also known as *Jensen’s contractive operator inequality*.

**Corollary** **1** **(Contractive version).** *Let f:I→R be a continuous function, and suppose that 0∈I. Then, f is operator convex on I and f(0)≤0 if and only if for some, hence, for every, n≥1 inequality (8) is valid for every n-tuple of bounded Hermitian operators $X_{1},\ldots ,X_{n}$ on an arbitrary Hilbert space H, with spectra contained in I, and every n-tuple of operators $\left(A_{1},\ldots ,A_{n}\right)$ on H satisfying $\sum_{i=1}^{n}A_{i}^{†}A_{i}\leq id_{H}.$*

The reason for the adjective ‘contractive’ can be easily understood by setting n=1; in this case, it follows that *f* is operator convex on an interval *I* containing 0 with f(0)≤0 if and only if

(9) $$f\left(A^{†}XA\right)\leq A^{†}f\left(X\right)A,$$

for every bounded Hermitian operator *X* with spectrum in *I* and every operator *A* such that $A^{†}A\leq id$, which implies that *A* is a contraction, i.e., ∥Ax∥≤∥x∥ for all x∈H or, equivalently, ∥A∥≤1.

Let us now consider two interacting quantum systems *a* and *b* with Hilbert state spaces $H_{a}$ and $H_{b}$, respectively. The associated composite quantum system has Hilbert state space $H_{ab}=H_{a}⊗H_{b}$.

In the following, we indicate with $X_{a},X_{b},X_{ab}$ generic operators of $B_{H}\left(H_{a}\right)$, $B_{H}\left(H_{b}\right)$, and $B_{H}\left(H_{ab}\right)$, respectively.

The *partial trace* ${\mathrm{Tr}}_{b}$ over subsystem *b* is a ‘superoperator’, i.e., a linear map ${\mathrm{Tr}}_{b}\in B\left(B_{H}\left(H_{ab}\right),B_{H}\left(H_{a}\right)\right)$, defined as the linear extension to the whole $B_{H}\left(H_{ab}\right)$ of the map

(10) $${\mathrm{Tr}}_{b}\left(X_{a}⊗X_{b}\right)=\mathrm{Tr}\left(X_{b}\right)X_{a}.$$

${\mathrm{Tr}}_{b}$ satisfies the following property:

(11) $$\mathrm{Tr}\left({\mathrm{Tr}}_{b}\left(X_{ab}\right)X_{a}\right)=\mathrm{Tr}\left(X_{ab}\left(X_{a}⊗id_{b}\right)\right).$$

See, e.g., [18], page 100.

The partial trace ${\mathrm{Tr}}_{b}$ is as follows:

- *Trace-preserving*: $\mathrm{Tr}\left(X_{ab}\right)=\mathrm{Tr}\left({\mathrm{Tr}}_{b}\left(X_{ab}\right)\right)$;
- *Positive*: if $X_{ab}\geq 0$, then ${\mathrm{Tr}}_{b}\left(X_{ab}\right)\geq 0$.

As a consequence, ${\mathrm{Tr}}_{b}$ maps states of $H_{ab}$ into states of $H_{a}$.

Moreover, ${\mathrm{Tr}}_{b}$ is *completely positive*; i.e., ${\mathrm{Tr}}_{b}⊗id_{H}$ is a positive linear map for all Hilbert spaces H. A trace-preserving and completely positive (or ‘CPTP’) linear map is called a *channel*.

The partial trace is actually one of the three main elements of every channel. In fact, thanks to the Stinespring theorem (see [19]), given any channel $C\in B(B_{H}\left(H_{a}\right),B_{H}\left(H_{a}\right))$, there exist an operator $Y\in B_{H}\left(H_{b}\right)$ and a unitary operator *U* on $H_{a}⊗H_{b}$ such that

(12) $$C\left(X\right):={\mathrm{Tr}}_{b}\left(U\left(X⊗Y\right)U^{†}\right),\;\forall X\in B_{H}\left(H_{a}\right).$$

By the Riesz representation theorem, we can define the adjoint of the partial trace ${\mathrm{Tr}}_{b}$ as the only operator ${\mathrm{Tr}}_{b}^{†}:B_{H}\left(H_{a}\right)\to B_{H}\left(H_{ab}\right)$ satisfying

(13) $${\langle X_{ab},{\mathrm{Tr}}_{b}^{†}\left(X_{a}\right)\rangle }_{B_{H}\left(H_{ab}\right)}={\langle {\mathrm{Tr}}_{b}\left(X_{ab}\right),X_{a}\rangle }_{B_{H}\left(H_{a}\right)}.$$

Writing the inner products explicitly and using property (11), we find

(14) $$\mathrm{Tr}\left(X_{ab}{\mathrm{Tr}}_{b}^{†}\left(X_{a}\right)\right)=\mathrm{Tr}\left({\mathrm{Tr}}_{b}\left(X_{ab}\right)X_{a}\right)=\mathrm{Tr}\left(X_{ab}\left(X_{a}⊗id_{b}\right)\right),$$

which allows us to write the explicit action of ${\mathrm{Tr}}_{b}^{†}$ as

(15) $${\mathrm{Tr}}_{b}^{†}\left(X_{a}\right)=X_{a}⊗id_{b}.$$

This formula implies that ${\left({\mathrm{Tr}}_{b}^{†}\left(X_{a}\right)\right)}^{†}={\mathrm{Tr}}_{b}^{†}\left(X_{a}^{†}\right)$ and that ${\mathrm{Tr}}_{b}^{†}$ is *unital*; i.e., it maps the identity of its domain to the identity of its range

(16) $${\mathrm{Tr}}_{b}^{†}\left(id_{a}\right)=id_{ab}.$$

Being a completely positive unital transformation, ${\mathrm{Tr}}_{b}^{†}$ is a so-called *Schwarz map* (see [20], Corollary 2.8); i.e., it satisfies the following inequality:

(17) $${\mathrm{Tr}}_{b}^{†}\left(X_{a}^{†}\right){\mathrm{Tr}}_{b}^{†}\left(X_{a}\right)\leq {\mathrm{Tr}}_{b}^{†}\left(X_{a}^{†}\;X_{a}\right).$$

This property is shared by the adjoint of any channel C.

### Relative Entropy in Quantum Information Theory

We devote a separate subsection to relative entropy, as its proper treatment entails the detailed development of several technical aspects.

This is essential for addressing certain subtleties that are often overlooked in the literature yet play a crucial role in the rigorous analysis of relative entropy.

Given any finite-dimensional Hilbert space H and any density operator $\rho \in B_{H}\left(H\right)$ with eigenvalues $\lambda_{j}\geq 0$, we indicate with Sp(ρ) its spectrum and with ${\mathrm{Sp}}_{+}\left(\rho \right)$ the subset of Sp(ρ) composed only of *strictly positive* eigenvalues.

It will be convenient to have the following index sets at hand: $I\left(\rho \right)=\{j\;:\;\lambda_{j}\in \mathrm{Sp}\left(\rho \right)\}$ and $I_{+}\left(\rho \right)=\left\{j\;:\;\lambda_{j}\in {\mathrm{Sp}}_{+}\left(\rho \right)\right\}$.

The *support* of ρ, denoted with supp(ρ), is the subset of H defined as follows:

(18) $$\begin{array}{cc} \mathrm{supp}(\rho ) & :=\mathrm{span}\{x_{j}\in H\;:\;x_{j}\;\mathrm{is}\;\mathrm{an}\;\mathrm{eigenvector}\;\mathrm{of}\;\rho \;\mathrm{with}\;\mathrm{eigenvalue}\;\lambda_{j}\in {\mathrm{Sp}}_{+}\left(\rho \right)\}\\ & =\underset{j\in I_{+}\left(\rho \right)}{⨁}E_{\lambda_{j}}, \end{array}$$

where $E_{\lambda_{j}}$ is the eigenspace relative to the eigenvalue $\lambda_{j}$.

From this definition and the spectral theorem for Hermitian operators, it immediately follows that

(19) supp(ρ)=ker(ρ)⊥=Im(ρ).

This implies that H=ker(ρ)⊕supp(ρ), so the positive definite operator $\rho {|}_{\mathrm{supp}(\rho )}$ has a trivial kernel; hence, it is invertible.

In particular, if ρ is positive definite, then ρ is invertible everywhere, and its image and support coincide with the entire H. On the other hand, non-invertible density operators, for instance, those corresponding to pure states, have support strictly included in H.

The deviation from purity, or mixedness, of ρ can be measured by its *von Neumann entropy*, defined as follows:

(20) S(ρ):=−Tr(ρlogρ).

The condition S(ρ)=0 is satisfied if and only if ρ is pure. In the literature, the precise definition of the logarithm of a matrix may vary according to the specific aims and context in which the matrix logarithm is employed.

For the purposes of this paper, the key property that the logarithm of a density operator ρ, or, more generally, of a Hermitian operator, must satisfy is exp(logρ)=ρ. For this reason, we adopt a definition of log via functional calculus in the extended real numbers with the following conventions:

(21) log(0)=−∞,exp(−∞)=0,0log0=0,

of course justified by the limits logε→−∞ as $\varepsilon \to 0^{+}$, $\exp\left(-M\right)\to 0^{+}$ as M→+∞, and εlogε→0 as $\varepsilon \to 0^{+}$.

Using the spectral theorem, if ρ is decomposed as

(22) $$\rho =\sum_{j\in I_{+}\left(\rho \right)}\lambda_{j}P_{j}+0P_{0},$$

where $P_{j}$ denotes the orthogonal projector onto the eigenspace $E_{\lambda_{j}}$, $j\in I_{+}\left(\rho \right)$, and $P_{0}$ is the orthogonal projector on ker(ρ), then, using the previous conventions, we have

(23) $$\log\rho :=\sum_{j\in I_{+}\left(\rho \right)}\log\left(\lambda_{j}\right)P_{j}+\left(-\infty \right)P_{0},$$

and so

(24) $$\exp\left(\log\rho \right)=\sum_{j\in I_{+}\left(\rho \right)}\exp\left(\log\lambda_{j}\right)P_{j}+\exp\left(-\infty \right)P_{0}=\sum_{j\in I_{+}\left(\rho \right)}\lambda_{j}P_{j}+0P_{0}=\rho .$$

Since the projectors satisfy the orthogonality relation $P_{i}P_{j}=\delta_{ij}P_{j}$ and by the convention 0log0=0, which accounts for the zero eigenvalue, we obtain

(25) $$S\left(\rho \right)=-\mathrm{Tr}\left(\rho \log\rho \right)=-\sum_{j\in I_{+}\left(\rho \right)}\lambda_{j}\log\left(\lambda_{j}\right)\mathrm{Tr}\left(P_{j}\right),$$

where $\mathrm{Tr}(P_{j})$ is the multiplicity of the eigenvalue $\lambda_{j}$, in the literature, it is actually more common to write just

(26) $$S\left(\rho \right)=-\sum_{j\in I_{+}\left(\rho \right)}\lambda_{j}\log\left(\lambda_{j}\right),$$

with the understanding that each eigenvalue is repeated according to its multiplicity.

The von Neumann entropy does not, by itself, tell us how one state differs from another. To capture the distinguishability of two states, the concept of *relative entropy* (sometimes called the *quantum Kullback–Leibler divergence*) must be introduced.

The definition of the relative entropy between two density operators ρ and σ is subjected to a condition on the compatibility of their supports, precisely the following:

(27) $$S\left(\rho ∥\sigma \right):=\left\{\begin{array}{cc} \mathrm{Tr}(\rho \log\rho -\rho \log\sigma ) & \mathrm{if}\;\mathrm{supp}(\rho )\subseteq \mathrm{supp}(\sigma )\\ +\infty & \mathrm{if}\;\mathrm{supp}(\rho )⊈\mathrm{supp}(\sigma ) \end{array}\right.,$$

which is known as the ‘*support-based definition*’ of relative entropy.

In order to understand why the condition supp(ρ)⊆supp(σ) is both necessary and sufficient for S(ρ∥σ) to be well-defined, we first note that

(28) Tr(ρlogρ−ρlogσ)=Tr(ρlogρ)−Tr(ρlogσ),

so, the first term of Formula (28) is minus the von Neumann entropy, which is always finite. In order to analyze the second term, let us consider, alongside Equation (22), the analogous spectral decomposition of σ

(29) $$\sigma =\sum_{k\in I_{+}\left(\sigma \right)}\mu_{k}\Pi_{k}+0\Pi_{0},$$

so that

(30) $$\log\sigma =\sum_{k\in I_{+}\left(\sigma \right)}\log\left(\mu_{k}\right)\Pi_{k}+\log\left(0\right)\Pi_{0},$$

where $\Pi_{k}$ is the orthogonal projector on the eigenspace relative to the positive eigenvalue $\mu_{k}$ of σ, and $\Pi_{0}$ is the projector on ker(σ). Then,

(31) $$\begin{array}{cc} \mathrm{Tr}(\rho \log\sigma ) & =\sum_{j\in I_{+}\left(\rho \right)}\sum_{k\in I_{+}\left(\sigma \right)}\lambda_{j}\log\left(\mu_{k}\right)\mathrm{Tr}\left(P_{j}\Pi_{k}\right)+\sum_{j\in I_{+}\left(\rho \right)}\lambda_{j}\log\left(0\right)\mathrm{Tr}\left(P_{j}\Pi_{0}\right), \end{array}$$

where the contribution of $P_{0}$ does not appear due to the convention 0log(0)=0.

The first term is always finite, since only strictly positive eigenvalues appear. Instead, the behavior of the second term depends on the value of $\mathrm{Tr}(P_{j}\Pi_{0})$. To compute it, let us consider any eigenbasis ${\left(x_{j}\right)}_{j\in I_{+}\left(\rho \right)}$ of supp(ρ) so that $P_{j}\left(x_{j}\right)=x_{j}$ for all $j\in I_{+}\left(\rho \right)$ and use the fact that orthogonal projectors are Hermitian and idempotent to write

(32) $$\begin{array}{cc} \mathrm{Tr}(P_{j}\Pi_{0}) & =\sum_{j\in I_{+}\left(\rho \right)}\langle x_{j},P_{j}\Pi_{0}x_{j}\rangle =\sum_{j\in I_{+}\left(\rho \right)}\langle P_{j}x_{j},\Pi_{0}x_{j}\rangle =\sum_{j\in I_{+}\left(\rho \right)}\langle x_{j},\Pi_{0}x_{j}\rangle \\ & =\sum_{j\in I_{+}\left(\rho \right)}\langle x_{j},\Pi_{0}\Pi_{0}x_{j}\rangle =\sum_{j\in I_{+}\left(\rho \right)}\langle \Pi_{0}x_{j},\Pi_{0}x_{j}\rangle =\sum_{j\in I_{+}\left(\rho \right)}{∥\Pi_{0}x_{j}∥}^{2}\geq 0. \end{array}$$

The second term in Equation (31) diverges to −∞ if and only if $\mathrm{Tr}(P_{j}\Pi_{0})>0$, i.e., when there exists at least one $j\in I_{+}\left(\rho \right)$ such that $x_{j}\in \mathrm{supp}\left(\rho \right)$ has a non-trivial projection onto ker(σ), i.e., $\Pi_{0}x_{j}$ is non-null, which is equivalent to saying that $x_{j}\notin \ker{\left(\sigma \right)}^{⊥}=\mathrm{supp}\left(\sigma \right)$. Therefore,

(33) $$\mathrm{Tr}\left(\rho \log\sigma \right)=-\infty \Leftrightarrow \mathrm{Tr}\left(P_{j}\Pi_{0}\right)>0\Leftrightarrow \mathrm{supp}\left(\rho \right)⊈\mathrm{supp}\left(\sigma \right),$$

which justifies the definition given in (27).

Instead, if supp(ρ)⊆supp(σ), then $\mathrm{Tr}(P_{j}\Pi_{0})=0$; additionally, using again the convention 0log(0)=0, the second term in Equation (31) vanishes, and we remain just with the first term, which can be written in an alternative form. Consider now the orthonormal eigenbases ${\left(x_{j}\right)}_{j\in J_{+}\left(\rho \right)}$, ${\left(y_{k}\right)}_{k\in I_{+}\left(\sigma \right)}$ of supp(ρ) and supp(σ), respectively; then, we have the following well-known formula (see, e.g., [21]):

(34) $$\mathrm{Tr}\left(P_{j}\Pi_{k}\right)={\left|\langle x_{j},y_{k}\rangle \right|}^{2},$$

which shows that the factor $\mathrm{Tr}(P_{j}\Pi_{k})$ codifies the transition probability between the pure states represented by the unit vectors $x_{j}$ and $y_{k}$.

In summary, when supp(ρ)⊆supp(σ), the relative entropy between ρ and σ can be written explicitly as follows:

(35) $$\begin{array}{cc} S(\rho ∥\sigma ) & =-S\left(\rho \right)-\sum_{j\in I_{+}\left(\rho \right)}\sum_{k\in I_{+}\left(\sigma \right)}\lambda_{j}\log\left(\mu_{k}\right)\mathrm{Tr}\left(P_{j}\Pi_{k}\right)\\ & =\sum_{j\in I_{+}\left(\rho \right)}\lambda_{j}\left(\log\left(\lambda_{j}\right)-\sum_{k\in I_{+}\left(\sigma \right)}\log\left(\mu_{k}\right)\;{\left|\langle x_{j},y_{k}\rangle \right|}^{2}\right). \end{array}$$

Two cases are particularly relevant in practical contexts:

- If ρ,σ>0, then their supports coincide with the entire Hilbert space H, and so their relative entropy is the finite real number given by Equation (35);
- Instead, if ρ>0 but σ is not, then their relative entropy is infinite. This happens, for instance, when ρ is a full-rank mixed state and σ is a pure state.

An equivalent and useful definition of the von Neumann and relative entropy appears in the literature (see, e.g., [22]). Rather than relying on the support of the density operators, this alternative definition is based on a regularization procedure.

Specifically, given a state ρ on H, one defines the regularized operator

(36) $$\rho_{\varepsilon }:=\rho +\varepsilon \;id_{H},$$

with ε>0. $\rho_{\varepsilon }$ is a positive definite Hermitian operator, and the spectral decompositions of $\rho_{\varepsilon }$ and $\log\rho_{\varepsilon }$ are

(37) $$\rho_{\varepsilon }=\sum_{j\in I(\rho )}\left(\lambda_{j}+\varepsilon \right)P_{j},\;\log\rho_{\varepsilon }=\sum_{j\in I(\rho )}\log\left(\lambda_{j}+\varepsilon \right)P_{j}.$$

We have

(38) $$\left(\lambda_{j}+\varepsilon \right)\log\left(\lambda_{j}+\varepsilon \right)\underset{\varepsilon \to 0^{+}}{⟶}\left\{\begin{array}{cc} \lambda_{j}\log\left(\lambda_{j}\right) & \mathrm{if}\;j\in I_{+}\left(\rho \right)\\ 0 & \mathrm{if}\;\lambda_{j}=0 \end{array}\right.,$$

so the von Neumann entropy of ρ is well-defined via the limit

(39) $$S\left(\rho \right):=-\lim_{\varepsilon \to 0^{+}}\mathrm{Tr}\left(\rho_{\varepsilon }\log\rho_{\varepsilon }\right).$$

Similarly, setting $\sigma_{\varepsilon }:=\sigma +\varepsilon \;id_{H}$, with ε>0, the relative entropy between ρ and σ can be defined as

(40) $$S\left(\rho ∥\sigma \right):=\lim_{\varepsilon \to 0^{+}}\mathrm{Tr}\left(\rho_{\varepsilon }\log\rho_{\varepsilon }-\rho_{\varepsilon }\log\sigma_{\varepsilon }\right),$$

known as the ‘*regularized definition*’ of relative entropy.

Let us verify that the regularized and support-based definitions of relative entropy coincide. Equation (40) splits into two terms; the first equals minus the regularized definition of the von Neumann entropy, which is finite by Equation (39), so the only issue to address concerns the second term of Equation (40).

For that, we write the spectral decomposition of $\sigma_{\varepsilon }$ as follows:

(41) $$\sigma_{\varepsilon }=\sum_{k\in I_{+}\left(\sigma \right)}\;\left(\mu_{k}+\varepsilon \right)\Pi_{k}+\varepsilon \Pi_{0}.$$

Repeating the same computations performed in the case of the support-based definition of S(ρ∥σ), we obtain

(42) $$\mathrm{Tr}\left(\rho_{\varepsilon }\log\sigma_{\varepsilon }\right)=\sum_{k\in I_{+}\left(\sigma \right)}\log\left(\mu_{k}+\varepsilon \right)\mathrm{Tr}\left(\rho_{\varepsilon }\Pi_{k}\right)+\log\left(\varepsilon \right)\mathrm{Tr}\left(\rho_{\varepsilon }\Pi_{0}\right).$$

If supp(ρ)⊆supp(σ), then $\mathrm{Tr}(\rho_{\varepsilon }\Pi_{0})\to 0$ when $\varepsilon \to 0^{+}$ and the last term in Equation (42) is absent, so the limit converges to the correct value.

Instead, if supp(ρ)⊈supp(σ), then $\mathrm{Tr}(\rho_{\varepsilon }\;\Pi_{0})\to \alpha >0$ when $\varepsilon \to 0^{+}$; consequently, the second term in Equation (42) diverges to −∞, thus matching the behavior of the support-based definition.

The relative entropy has several important properties (see, e.g., [23]):

- *Klein’s inequality*: S(ρ∥σ)≥0 for all ρ,σ, and S(ρ∥σ)=0 if and only if ρ=σ. This property motivates the reason why the relative entropy, despite lacking symmetry in its arguments, is taken to be a measure of the distinguishability of states in quantum theories.
- *Invariance under unitary conjugation*: $S\left(U\rho U^{†}∥U\sigma U^{†}\right)=S\left(\rho ∥\sigma \right)$ for all unitary operators *U* acting on the same Hilbert space as ρ and σ.
- *Additivity w.r.t. tensor product*: $S\left(\rho_{1}⊗\rho_{2}∥\sigma_{1}⊗\sigma_{2}\right)=S\left(\rho_{1}∥\sigma_{1}\right)+S\left(\rho_{2}∥\sigma_{2}\right)$ for all density operators $\sigma_{j},\rho_{j},j=1,2$.

The *monotonicity of S under partial trace* is represented by the inequality

(43) $$S({\mathrm{Tr}}_{b}\left(\rho \right)∥{\mathrm{Tr}}_{b}\left(\sigma \right))\leq S\left(\rho ∥\sigma \right),$$

which, together with Stinespring’s theorem and the three previously mentioned properties of *S*, permits to prove that quantum distinguishability does not increase under the action of a generic channel C

(44) S(C(ρ)∥C(σ))≤S(ρ∥σ),

a formula also known as the *data processing inequality* (DPI). In fact,

(45) $$\begin{array}{cc} S(C(\rho )∥C(\sigma )) & =S({\mathrm{Tr}}_{b}\left(U\left(\rho ⊗X\right)U^{†}\right)\;∥\;{\mathrm{Tr}}_{b}\left(U\left(\sigma ⊗X\right)U^{†}\right))\\ & \leq S(U\left(\rho ⊗X\right)U^{†}∥U\left(\sigma ⊗X\right)U^{†})\\ & =S(\rho ⊗X∥\sigma ⊗X)=S(\rho ∥\sigma )+S(X,X)\\ & =S(\rho ∥\sigma ). \end{array}$$

Inequality (44) explains why, in the quantum information literature, a channel is often referred to as a *coarse-graining* procedure, a term borrowed from statistical mechanics.

This terminology reflects the idea that information about different quantum states is lost through the action of the channel, as previously distinguishable states may become indistinguishable after the transformation.

While the first three properties of *S* mentioned above are relatively straightforward to prove, its monotonicity under partial trace is considerably more subtle. In the next two sections, we provide a detailed analysis of the proof originally proposed by Petz and Uhlmann.

## 3. Petz’s Proof of the Monotonicity of the Relative Entropy Under Partial Trace

In this section, we examine the strategy proposed by Petz in [10] for proving the monotonicity of relative entropy under the partial trace operation, which is based on a clever reformulation of the expression of the relative entropy as a suitable inner product inspired by an analogous construction by Araki [2].

We will show that Petz’s proof is flawed due to an incorrect application of the contractive version of Jensen’s operator inequality. We will explain how this issue can be circumvented, thus restoring the validity of Petz’s overall approach. Furthermore, we will show how to extend it to also incorporate non-invertible density operators.

The notation that will be used throughout this section is detailed below:

- Given the finite-dimensional Hilbert spaces $\left(H_{a},{\langle \;,\;\rangle }_{a}\right)$ and $\left(H_{b},{\langle \;,\;\rangle }_{b}\right)$, we define $H_{ab}:=H_{a}⊗H_{b}$, with inner product ${\langle \;,\;\rangle }_{ab}$ induced by those of $H_{a}$ and $H_{b}$;
- Operators of $B_{H}\left(H_{a}\right)$ will be denoted as X,Y,Z, and operators of $B_{H}\left(H_{ab}\right)$ will be denoted by A,B,C;
- $\rho ,\sigma \in B_{H}\left(H_{ab}\right)$ are two positive definite (invertible) density operators (actually, for the following analysis, only ρ has to be invertible; however, as we noted with the support-based definition of the relative entropy, if ρ>0, then its support is the entire H, and so, for our analysis to be meaningful, we also have to demand σ>0): ρ,σ>0.

In order to rewrite the relative entropy as an inner product, we must introduce some suitable superoperators.

Precisely, for fixed $B,C\in B_{H}\left(H_{ab}\right)$, B>0, consider the Hermitian superoperators $R_{B},L_{C},\Delta_{B,C}\in B_{H}\left(B_{H}\left(H_{ab}\right)\right)$ given by the right and left operator multiplications and the relative modular operator, respectively, i.e.,

(46) $$R_{B}\left(A\right):=AB,\;L_{C}\left(A\right):=CA,\;\Delta_{B,C}\left(A\right):=L_{C}\circ {R_{B}}^{-1}\left(A\right)=CAB^{-1}.$$

Clearly, ${R_{B}}^{-1}=R_{B^{-1}}$, ${L_{C}}^{-1}=L_{C^{-1}}$, and

(47) $$\left[L_{C},R_{B}\right]=\left[L_{C},{R_{B}}^{-1}\right]=0.$$

All these superoperators are Hermitian; in fact,

(48) $${\langle R_{B}\left(A\right),C\rangle }_{ab}=\mathrm{Tr}\left({\left(AB\right)}^{†}C\right)=\mathrm{Tr}\left(BAC\right)=\mathrm{Tr}\left(ACB\right)={\langle A,R_{B}\left(C\right)\rangle }_{ab},$$

and similarly for $L_{C}$. Regarding $\Delta_{B,C}$, using Equation (47), we have

(49) $$\Delta_{B,C}^{†}={\left(L_{C}{R_{B}}^{-1}\right)}^{†}={\left({R_{B}}^{-1}\right)}^{†}L_{C}={\left(R_{B}^{†}\right)}^{-1}L_{C}=R_{B}^{-1}\circ L_{C}=L_{C}\circ {R_{B}}^{-1}=\Delta_{B,C}.$$

If we consider the specific case in which A=ρ>0 and B=σ>0, then we obtain

(50) $$\begin{array}{cc} \log(\Delta_{\rho ,\sigma }) & =\log\left(L_{\sigma }\circ R_{\rho }^{-1}\right)=\log(\exp\left(\log\left(L_{\sigma }\right)\right)\exp\left(\log\left(R_{\rho }^{-1}\right)\right)\\ & =\log\left(\exp\left(\log\left(L_{\sigma }\right)-\log\left(R_{\rho }\right)\right)\right)=\log\left(L_{\sigma }\right)-\log\left(R_{\rho }\right). \end{array}$$

Since $\log\left(L_{\sigma }\right)=L_{\log(\sigma )}$ and $\log\left(R_{\rho }\right)=R_{\log(\rho )}$, applying formula (50) to $\rho^{1/2}$ gives

(51) $$\log\left(\Delta_{\rho ,\sigma }\right)\left(\rho^{1/2}\right)=\log\left(\sigma \right)\rho^{1/2}-\rho^{1/2}\log\left(\rho \right).$$

This last identity, the cyclic property of the trace, and the property $[\rho^{1/2},\log\left(\rho \right)]=0$ imply that

(52) $$\begin{array}{cc} S(\rho ∥\sigma ) & =\mathrm{Tr}\left[\rho \log\left(\rho \right)-\rho \log\left(\sigma \right)\right]=\mathrm{Tr}[\rho^{1/2}\left(\rho^{1/2}\log\left(\rho \right)-\rho^{1/2}\log\left(\sigma \right)\right)]\\ & =\mathrm{Tr}[\left(\rho^{1/2}\log\left(\rho \right)-\rho^{1/2}\log\left(\sigma \right)\right)\rho^{1/2}]\\ & =\mathrm{Tr}[\rho^{1/2}\log\left(\rho \right)\rho^{1/2}-\rho^{1/2}\log\left(\sigma \right)\rho^{1/2}]\\ & =\mathrm{Tr}[\rho^{1/2}\rho^{1/2}\log\left(\rho \right)-\rho^{1/2}\log\left(\sigma \right)\rho^{1/2}]\\ & =-\mathrm{Tr}[\rho^{1/2}\left(\log\left(\sigma \right)\rho^{1/2}-\rho^{1/2}\log\left(\rho \right)\right)]\\ & =-{\langle \rho^{1/2},\log\left(\Delta_{\rho ,\sigma }\right)\left(\rho^{1/2}\right)\rangle }_{ab}, \end{array}$$

which is the ‘inner product reformulation’ of the relative entropy between the positive definite states ρ and σ that we were searching for.

We can obtain an analogous formula for the relative entropy of the partial traces of ρ and σ. To this end, if we fix $Y,Z\in B_{H}\left(H_{a}\right)$, Y>0, then we can define the Hermitian superoperators $R_{Y}^{a},L_{Z}^{a},\Delta_{\rho ,\sigma }^{a}\in B_{H}\left(B_{H}\left(H_{a}\right)\right)$ as follows:

(53) $$R_{Y}^{a}\left(X\right):=XY,\;L_{Z}^{a}\left(X\right):=ZX,\;\Delta_{Y,Z}^{a}\left(X\right):=L_{Z}\circ {R_{Y}}^{-1}\left(X\right)=ZXY^{-1}.$$

By carrying out computations analogous to those in Equation (52) but this time using the superoperators $R_{{\mathrm{Tr}}_{b}\left(\rho \right)}^{a},L_{{\mathrm{Tr}}_{b}\left(\sigma \right)}^{a},\Delta_{\rho ,\sigma }^{a}:=L_{{\mathrm{Tr}}_{b}\left(\sigma \right)}^{a}\circ R_{{\mathrm{Tr}}_{b}{\left(\rho \right)}^{-1}}^{a}$, we obtain

(54) $$S\left({\mathrm{Tr}}_{b}\left(\rho \right)∥{\mathrm{Tr}}_{b}\left(\sigma \right)\right)=-{\langle {\mathrm{Tr}}_{b}{\left(\rho \right)}^{1/2},\log\left(\Delta_{\rho ,\sigma }^{a}\right)\;\left({\mathrm{Tr}}_{b}{\left(\rho \right)}^{1/2}\right)\rangle }_{a}\;.$$

Due to the minus sign in front of the inner products appearing in Equations (52) and (54), we have that *the monotonicity of the relative entropy under partial trace is equivalent to*

(55) $${\langle \rho^{1/2},\log\left(\Delta_{\rho ,\sigma }\right)\left(\rho^{1/2}\right)\rangle }_{ab}\leq {\langle {\mathrm{Tr}}_{b}{\left(\rho \right)}^{1/2},\log\left(\Delta_{\rho ,\sigma }^{a}\right)\left({\mathrm{Tr}}_{b}{\left(\rho \right)}^{1/2}\right)\rangle }_{a}\;.$$

These inner products are defined on two different Hilbert spaces, $B_{H}\left(H_{ab}\right)$ and $B_{H}\left(H_{a}\right)$; in order to perform a meaningful comparison and prove the inequality, Petz introduced a superoperator $V_{\rho }:B_{H}\left(H_{a}\right)\to B_{H}\left(H_{ab}\right)$ through the explicit formula

(56) $$V_{\rho }\left(X\;{\mathrm{Tr}}_{b}{\left(\rho \right)}^{1/2}\right):={\mathrm{Tr}}_{b}^{†}\left(X\right)\;\rho^{1/2},$$

which serves as a bridge between the reduced state ${\mathrm{Tr}}_{b}\left(\rho \right)$ and the full state ρ. While, as we are going to show, this definition is computationally effective, it may at first appear somewhat ad hoc. Actually, a seemingly more natural choice for $V_{\rho }$ would have been ${\mathrm{Tr}}_{b}^{†}$ for two reasons: first, as for $V_{\rho }$, ${\mathrm{Tr}}_{b}^{†}$ is a map between $B_{H}\left(H_{a}\right)$ and $B_{H}\left(H_{ab}\right)$, and, second, it satisfies the Schwarz inequality (17), which, in the following, will play a crucial role in the proof of inequality (55).

It turns out that $V_{\rho }$ is tightly related to the adjoint of the partial trace ${\mathrm{Tr}}_{b}$, not w.r.t. the original inner products of $H_{a}$ and $H_{ab}$, but w.r.t. suitably *weighted inner products* that naturally emerge from the previous structural analysis of the relative entropy in terms of superoperators.

In fact, the reformulations of the relative entropy obtained in Equations (52) and (54) involve inner products weighted by $\rho^{1/2}$ and ${\mathrm{Tr}}_{b}{\left(\rho \right)}^{1/2}$, respectively. This observation suggests that the correct notion of adjoint to consider for ${\mathrm{Tr}}_{b}$ is the one defined relative to the inner products (the positive definiteness of these inner products is guaranteed by the fact that $\rho ,{\mathrm{Tr}}_{b}\left(\rho \right)>0$)

(57) $${\langle A,B\rangle }_{ab,\;\rho }:={\langle R_{\rho^{-1/2}}\left(A\right),B\rangle }_{ab},\;{\langle X,Y\rangle }_{a,\;\rho }:={\langle R_{{\mathrm{Tr}}_{b}{\left(\rho \right)}^{-1/2}}^{a}\left(X\right),Y\rangle }_{a}.$$

We have

(58) $$\begin{array}{cc} {\langle X,{\mathrm{Tr}}_{b}\left(A\right)\rangle }_{a,\;\rho } & ={\langle R_{{\mathrm{Tr}}_{b}{\left(\rho \right)}^{-1/2}}^{a}\left(X\right),{\mathrm{Tr}}_{b}\left(A\right)\rangle }_{a}={\langle {\mathrm{Tr}}_{b}^{†}\circ R_{{\mathrm{Tr}}_{b}{\left(\rho \right)}^{-1/2}}^{a}\left(X\right),A\rangle }_{ab}\\ & ={\langle R_{\rho^{-1/2}}\circ R_{\rho^{1/2}}\circ {\mathrm{Tr}}_{b}^{†}\circ R_{{\mathrm{Tr}}_{b}{\left(\rho \right)}^{-1/2}}^{a}\left(X\right),A\rangle }_{ab}\\ & ={\langle R_{\rho^{1/2}}\circ {\mathrm{Tr}}_{b}^{†}\circ R_{{\mathrm{Tr}}_{b}{\left(\rho \right)}^{-1/2}}^{a}\left(X\right),A\rangle }_{ab,\;\rho }. \end{array}$$

So, the adjoint of ${\mathrm{Tr}}_{b}$ w.r.t. the weighted inner products defined above is the operator ${\mathrm{Tr}}_{b}^{†,\;\rho }:=R_{\rho^{1/2}}\circ {\mathrm{Tr}}_{b}^{†}\circ R_{{\mathrm{Tr}}_{b}{\left(\rho \right)}^{-1/2}}^{a}$, which, for all $X\in B_{H}\left(H_{a}\right)$, satisfies

(59) $$\left(R_{\rho^{1/2}}\circ {\mathrm{Tr}}_{b}^{†}\circ R_{{\mathrm{Tr}}_{b}{\left(\rho \right)}^{-1/2}}^{a}\right)\left(X\;{\mathrm{Tr}}_{b}{\left(\rho \right)}^{1/2}\right)=\left(R_{\rho^{1/2}}\circ {\mathrm{Tr}}_{b}^{†}\right)\left(X\right)={\mathrm{Tr}}_{b}^{†}\left(X\right)\;\rho^{1/2},$$

and, therefore, $V_{\rho }={\mathrm{Tr}}_{b}^{†,\;\rho }$; i.e.,

(60) $$V_{\rho }=R_{\rho^{1/2}}\circ {\mathrm{Tr}}_{b}^{†}\circ R_{{\mathrm{Tr}}_{b}{\left(\rho \right)}^{-1/2}}^{a}.$$

From this fact, we obtain that the explicit action of $V_{\rho }$ on any $X\in B_{H}\left(H_{a}\right)$ is

(61) $$V_{\rho }\left(X\right)=\left(X\;{\mathrm{Tr}}_{b}{\left(\rho \right)}^{-1/2}⊗id_{b}\right)\;\rho^{1/2},$$

and this implies immediately that $V_{\rho }$ transforms ${\mathrm{Tr}}_{b}{\left(\rho \right)}^{1/2}$ into $\rho^{1/2}$:

(62) $$V_{\rho }\left({\mathrm{Tr}}_{b}{\left(\rho \right)}^{1/2}\right)=\rho^{1/2}.$$

Repeatedly using the cyclic property of the trace and Schwarz’s inequality (17) satisfied by ${\mathrm{Tr}}_{b}^{†}$, we can prove that $V_{\rho }$ is a contraction

(63) $$\begin{array}{cc} ∥V_{\rho }\left(X{\mathrm{Tr}}_{b}{\left(\rho \right)}^{1/2}\right){∥}^{2} & ={\langle {\mathrm{Tr}}_{b}^{†}\left(X\right)\rho^{1/2},{\mathrm{Tr}}_{b}^{†}\left(X\right)\rho^{1/2}\rangle }_{ab}=\mathrm{Tr}\left[{\left({\mathrm{Tr}}_{b}^{†}\left(X\right)\rho^{1/2}\right)}^{†}{\mathrm{Tr}}_{b}^{†}\left(X\right)\rho^{1/2}\right]\\ & =\mathrm{Tr}\left[\rho^{1/2}{\mathrm{Tr}}_{b}^{†}\left(X^{†}\right){\mathrm{Tr}}_{b}^{†}\left(X\right)\rho^{1/2}\right]=\mathrm{Tr}\left[{\mathrm{Tr}}_{b}^{†}\left(X^{†}\right){\mathrm{Tr}}_{b}^{†}\left(X\right)\rho \right]\\ & \leq \mathrm{Tr}\left[{\mathrm{Tr}}_{b}^{†}\left(X^{†}X\right)\rho \right]={\langle {\mathrm{Tr}}_{b}^{†}\left(X^{†}X\right),\rho \rangle }_{ab}={\langle X^{†}X,{\mathrm{Tr}}_{b}\left(\rho \right)\rangle }_{a}\\ & =\mathrm{Tr}\left(X^{†}X{\mathrm{Tr}}_{b}{\left(\rho \right)}^{1/2}{\mathrm{Tr}}_{b}{\left(\rho \right)}^{1/2}\right)=\mathrm{Tr}\left[{\mathrm{Tr}}_{b}{\left(\rho \right)}^{1/2}X^{†}X{\mathrm{Tr}}_{b}{\left(\rho \right)}^{1/2}\right]\\ & =\mathrm{Tr}\left[{\left(X{\mathrm{Tr}}_{b}{\left(\rho \right)}^{1/2}\right)}^{†}X{\mathrm{Tr}}_{b}{\left(\rho \right)}^{1/2}\right]={\langle X{\mathrm{Tr}}_{b}{\left(\rho \right)}^{1/2},X{\mathrm{Tr}}_{b}{\left(\rho \right)}^{1/2}\rangle }_{a}\\ & =∥X{\mathrm{Tr}}_{b}{\left(\rho \right)}^{1/2}{∥}^{2}. \end{array}$$

Note now that

(64) $$\begin{array}{cc} {\langle \Delta_{\rho ,\sigma }^{a}\left(X\right){\mathrm{Tr}}_{b}{\left(\rho \right)}^{1/2},X{\mathrm{Tr}}_{b}{\left(\rho \right)}^{1/2}\rangle }_{a} & ={\langle {\mathrm{Tr}}_{b}\left(\sigma \right)X{\mathrm{Tr}}_{b}{\left(\rho \right)}^{-1/2},X{\mathrm{Tr}}_{b}{\left(\rho \right)}^{1/2}\rangle }_{a}\\ & =\mathrm{Tr}[{\left({\mathrm{Tr}}_{b}\left(\sigma \right)X{\mathrm{Tr}}_{b}{\left(\rho \right)}^{-1/2}\right)}^{†}X{\mathrm{Tr}}_{b}{\left(\rho \right)}^{1/2}]\\ & =\mathrm{Tr}[{\mathrm{Tr}}_{b}{\left(\rho \right)}^{-1/2}X^{†}{\mathrm{Tr}}_{b}\left(\sigma \right)X{\mathrm{Tr}}_{b}{\left(\rho \right)}^{1/2}]\\ & =\mathrm{Tr}[XX^{†}{\mathrm{Tr}}_{b}\left(\sigma \right)]. \end{array}$$

Using the equality just proven, we can show that $V_{\rho }^{†}\Delta_{\rho ,\sigma }V_{\rho }\leq \Delta_{\rho ,\sigma }^{a}$; in fact,

(65) $$\begin{array}{cc} {\langle V_{\rho }^{†}\Delta_{\rho ,\sigma }V_{\rho }\left(X{\mathrm{Tr}}_{b}{\left(\rho \right)}^{1/2}\right),X{\mathrm{Tr}}_{b}{\left(\rho \right)}^{1/2}\rangle }_{a} & ={\langle \Delta_{\rho ,\sigma }V_{\rho }\left(X{\mathrm{Tr}}_{b}{\left(\rho \right)}^{1/2}\right),V_{\rho }\left(X{\mathrm{Tr}}_{b}{\left(\rho \right)}^{1/2}\right)\rangle }_{ab}\\ & ={\langle \Delta_{\rho ,\sigma }\left({\mathrm{Tr}}_{b}^{†}\left(X\right)\rho^{1/2}\right),{\mathrm{Tr}}_{b}^{†}\left(X\right)\rho^{1/2}\rangle }_{ab}\\ & =\langle \sigma {\mathrm{Tr}}_{b}^{†}\left(X\right)\rho^{-1/2},{\mathrm{Tr}}_{b}^{†}\left(X\right)\rho^{1/2}\rangle \\ & =\mathrm{Tr}[\rho^{-1/2}{\mathrm{Tr}}_{b}^{†}\left(X^{†}\right)\sigma {\mathrm{Tr}}_{b}^{†}\left(X\right)\rho^{1/2}]\\ & =\mathrm{Tr}[{\mathrm{Tr}}_{b}^{†}\left(X\right){\mathrm{Tr}}_{b}^{†}\left(X^{†}\right)\sigma ]\\ & \leq \mathrm{Tr}\left[{\mathrm{Tr}}_{b}^{†}\left(XX^{†}\right)\sigma \right]={\langle {\mathrm{Tr}}_{b}^{†}\left(XX^{†}\right),\sigma \rangle }_{ab}\\ & ={\langle XX^{†},{\mathrm{Tr}}_{b}\left(\sigma \right)\rangle }_{a}=\mathrm{Tr}\left[XX^{†}{\mathrm{Tr}}_{b}\left(\sigma \right)\right]\\ & ={\langle \Delta_{\rho ,\sigma }^{a}\left(X\right){\mathrm{Tr}}_{b}{\left(\rho \right)}^{1/2},X{\mathrm{Tr}}_{b}{\left(\rho \right)}^{1/2}\rangle }_{a}, \end{array}$$

where we again used Schwarz’s inequality, and, to write the last equality, we applied Equation (64).

Since log(x) is an operator monotone function, the inequality $V_{\rho }^{†}\Delta_{\rho ,\sigma }V_{\rho }\leq \Delta_{\rho ,\sigma }^{a}$ implies

(66) $$\log\left(V_{\rho }^{†}\Delta_{\rho ,\sigma }V_{\rho }\right)\leq \log\left(\Delta_{\rho ,\sigma }^{a}\right),$$

and so

(67) $$\begin{array}{cc} S({\mathrm{Tr}}_{b}\left(\rho \right)∥{\mathrm{Tr}}_{b}\left(\sigma \right)) & ={\langle {\mathrm{Tr}}_{b}{\left(\rho \right)}^{1/2},-\log\left(\Delta_{\rho ,\sigma }^{a}\right)\left({\mathrm{Tr}}_{b}{\left(\rho \right)}^{1/2}\right)\rangle }_{a}\\ & \leq {\langle {\mathrm{Tr}}_{b}{\left(\rho \right)}^{1/2},-\log\left(V_{\rho }^{†}\Delta_{\rho ,\sigma }V_{\rho }\right)\left({\mathrm{Tr}}_{b}{\left(\rho \right)}^{1/2}\right)\rangle }_{a}. \end{array}$$

Petz’s strategy to make the relative entropy S(ρ∥σ) appear on the right-hand side of the previous inequality consists of using the fact that $V_{\rho }$ is a contraction to apply the contractive Jensen operator inequality (9) with f(x)=−log(x). In this way, due to (62), we would get

(68) $$\begin{array}{cc} S({\mathrm{Tr}}_{b}\left(\rho \right)∥{\mathrm{Tr}}_{b}\left(\sigma \right)) & \leq {\langle {\mathrm{Tr}}_{b}{\left(\rho \right)}^{1/2},-\log\left(V_{\rho }^{†}\Delta_{\rho ,\sigma }V_{\rho }\right)\left({\mathrm{Tr}}_{b}{\left(\rho \right)}^{1/2}\right)\rangle }_{a}\\ & \leq {\langle {\mathrm{Tr}}_{b}{\left(\rho \right)}^{1/2},V_{\rho }^{†}\left(-\log\left(\Delta_{\rho ,\sigma }\right)\right)V_{\rho }\left({\mathrm{Tr}}_{b}{\left(\rho \right)}^{1/2}\right)\rangle }_{a}\\ & ={\langle V_{\rho }{\mathrm{Tr}}_{b}{\left(\rho \right)}^{1/2},-\log\left(\Delta_{\rho ,\sigma }\right)V_{\rho }\left({\mathrm{Tr}}_{b}{\left(\rho \right)}^{1/2}\right)\rangle }_{ab}\\ & ={\langle \rho^{1/2},-\log\left(\Delta_{\rho ,\sigma }\right)\left(\rho^{1/2}\right)\rangle }_{ab}\\ & =S(\rho ∥\sigma ), \end{array}$$

and so the proof of the monotonicity of *S* w.r.t. the partial trace ${\mathrm{Tr}}_{b}$ would be achieved.

For this argument to be valid, −log(x) is required to be operator convex, which is true, to be well-defined at x=0 and to satisfy −log(0)≤0.

Petz circumvented the lack of definition of −log(x) in x=0 by using the following integral identity:

(69) $$\begin{array}{cc} \int_{0}^{+\infty }\left(\frac{1}{x+\xi }-\frac{1}{1+\xi }\right)d\xi & =\lim_{M\to +\infty }{\left.\left(\log(x+\xi )-\log(1+\xi )\right)\right|}_{0}^{M}=\\ \; & =\lim_{M\to +\infty }\log\left(\frac{x+M}{1+M}\right)-\log\left(x\right)\\ & =-\log(x). \end{array}$$

Since the integral over [0,+∞) coincides with that over (0,+∞), we may restrict our attention to strictly positive values of ξ. If we denote by $id_{ab}$ the identity operator on $B_{H}\left(H_{ab}\right)$, we have

(70) $$\Delta_{\rho ,\sigma ,\xi }\equiv {\left(\Delta_{\rho ,\sigma }+\xi id_{ab}\right)}^{-1}-{\left(id_{ab}+\xi id_{ab}\right)}^{-1}={\left(\Delta_{\rho ,\sigma }+\xi id_{ab}\right)}^{-1}-id_{ab}{\left(1+\xi \right)}^{-1},$$

moreover,

(71) $${\langle \rho^{1/2},-id_{ab}{\left(1+\xi \right)}^{-1}\rho^{1/2}\rangle }_{ab}=-{\left(1+\xi \right)}^{-1}\mathrm{Tr}\left(\rho \right)=-{\left(1+\xi \right)}^{-1},$$

thus,

(72) $${\langle \rho^{1/2},\Delta_{s,t,\xi }\;\rho^{1/2}\rangle }_{ab}={\langle \rho^{1/2},{\left(\Delta_{\rho ,\sigma }+\xi id_{ab}\right)}^{-1}\rho^{1/2}\rangle }_{ab}-{\left(1+\xi \right)}^{-1}.$$

It follows that

(73) $$S\left(\rho ∥\sigma \right)=\int_{0}^{\infty }\left({\langle \rho^{1/2},{\left(\Delta_{\rho ,\sigma }+\xi id_{ab}\right)}^{-1}\rho^{1/2}\rangle }_{ab}-{\left(1+\xi \right)}^{-1}\right)d\xi ,$$

and, analogously,

(74) $$S\left({\mathrm{Tr}}_{b}\left(\rho \right)∥{\mathrm{Tr}}_{b}\left(\sigma \right)\right)=\int_{0}^{\infty }\left({\langle {\mathrm{Tr}}_{b}{\left(\rho \right)}^{1/2},{\left(\Delta_{\rho ,\sigma }^{a}+\xi id_{a}\right)}^{-1}{\mathrm{Tr}}_{b}{\left(\rho \right)}^{1/2}\rangle }_{a}-{\left(1+\xi \right)}^{-1}\right)d\xi .$$

For all ξ∈(0,+∞), the function $g_{\xi }$ given by $x\mapsto {\left(x+\xi \right)}^{-1}-{\left(1+\xi \right)}^{-1}$ is well-defined for x=0, and it is operator convex and operator monotone (decreasing); see [16] chapter V.1. Thanks to this last property, we have

(75) $${\left(\Delta_{\rho ,\sigma }^{a}+\xi \right)}^{-1}\leq {\left(V_{\rho }^{†}\Delta_{\rho ,\sigma }^{a}V_{\rho }+\xi \right)}^{-1}.$$

However, $g_{\xi }\left(0\right)=\xi^{-1}-{\left(1+\xi \right)}^{-1}>0$ for all ξ∈(0,+∞), so *the contractive Jensen operator inequality cannot be used to write*

(76) $${\left(V_{\rho }^{†}\Delta_{\rho ,\sigma }^{a}V_{\rho }+\xi \right)}^{-1}\leq V_{\rho }^{†}{\left(\Delta_{\rho ,\sigma }^{a}+\xi \right)}^{-1}V_{\rho },$$

which would lead to the proof of the monotonicity of the relative entropy with computations analogous to those shown in Formula (68).

**Figure 1:**
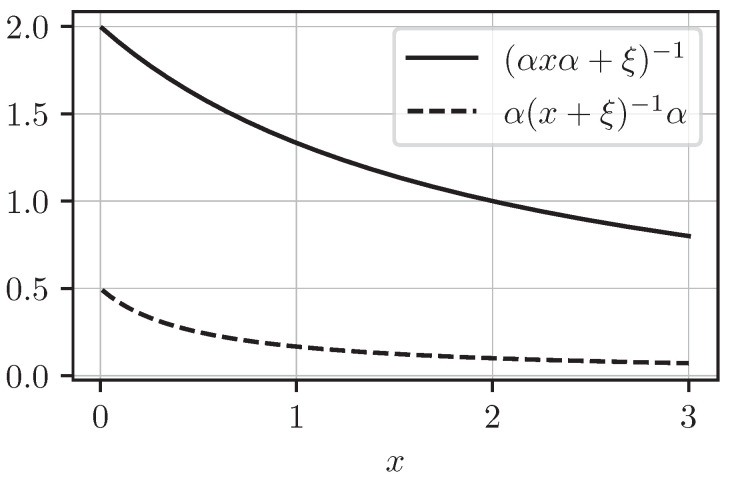
Comparison of ${\left(\alpha x\alpha +\xi \right)}^{-1}$ and $\alpha {\left(x+\xi \right)}^{-1}\alpha$, illustrating the failure of the Jensen-type inequality in the scalar case, with α=0.5 and ξ=0.5.

A simple counterexample illustrating the failure of inequality (76) arises in the scalar case, where a contraction reduces to a multiplication by a real coefficient α∈(0,1] and satisfies $\alpha^{†}=\alpha$. The inequality

(77) $${\left(\alpha x\alpha +\xi \right)}^{-1}\leq \alpha {\left(x+\xi \right)}^{-1}\alpha ,$$

is *false* for all ξ∈(0,+∞), as shown in Figure 1.

**Figure 2:**
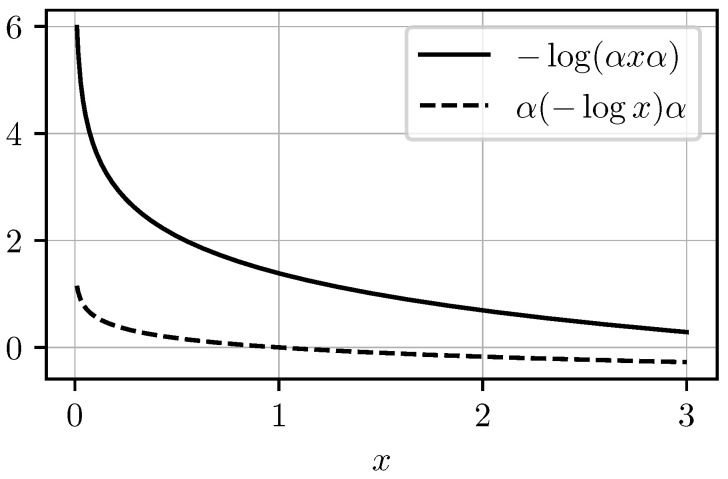
Counterexample showing that the inequality −log(αxα)≤−αlog(x)α fails for α=0.5.

Note that the inequality $-\log\left(V^{†}XV\right)\leq -V^{†}\log\left(X\right)V$ is also false for a generic contraction *V*, as we show in Figure 2 with a counterexample in the scalar case.

To summarize, showing that $V_{\rho }$ is a contraction does not permit the use of the contractive version of Jensen’s operator inequality to prove the monotonicity of the relative entropy.

### 3.1. Correction of Petz’s Strategy to Prove the Relative Entropy Monotonicity

There is a simple correction of Petz’s strategy that restores the validity of his approach to prove the monotonicity of the relative entropy in a rigorous way. The correction was provided by Petz himself, together with Nielsen, in [24]. The same line of reasoning can also be found in [25,26].

The key idea of the correction lies in recognizing that the operator $V_{\rho }$ is not merely a contraction but an isometry; i.e., $V_{\rho }^{†}V_{\rho }$ is the identity operator on $B_{H}\left(H_{a}\right)$. In fact, using Equations (11) and (56), we have

(78) $$\begin{array}{cc} {\langle Y{\mathrm{Tr}}_{b}{\left(\rho \right)}^{1/2},V_{\rho }^{†}V_{\rho }\left(X{\mathrm{Tr}}_{b}{\left(\rho \right)}^{1/2}\right)\rangle }_{a} & ={\langle V_{\rho }\left(Y{\mathrm{Tr}}_{b}{\left(\rho \right)}^{1/2}\right),V_{\rho }\left(X{\mathrm{Tr}}_{b}{\left(\rho \right)}^{1/2}\right)\rangle }_{ab}\\ \; & ={\langle {\mathrm{Tr}}_{b}^{†}\left(Y\right)\rho^{1/2},{\mathrm{Tr}}_{b}^{†}\left(X\right)\rho^{1/2}\rangle }_{ab}\\ \; & =\mathrm{Tr}[\left(Y^{†}⊗id_{b}\right)\left(X⊗id_{b}\right)\rho ]\\ \; & =\mathrm{Tr}\left[\left(Y^{†}X⊗id_{b}\right)\rho \right]=\mathrm{Tr}\left[Y^{†}X{\mathrm{Tr}}_{b}\left(\rho \right)\right]\\ \; & =\mathrm{Tr}[{\mathrm{Tr}}_{b}{\left(\rho \right)}^{1/2}Y^{†}X{\mathrm{Tr}}_{b}{\left(\rho \right)}^{1/2}]\\ \; & =\mathrm{Tr}[{\left(Y{\mathrm{Tr}}_{b}{\left(\rho \right)}^{1/2}\right)}^{†}X{\mathrm{Tr}}_{b}{\left(\rho \right)}^{1/2}]\\ \; & ={\langle Y{\mathrm{Tr}}_{b}{\left(\rho \right)}^{1/2},X{\mathrm{Tr}}_{b}{\left(\rho \right)}^{1/2}\rangle }_{a}. \end{array}$$

This allows us to apply point (iii) of Theorem 1 with $H=B_{H}\left(H_{ab}\right)$, $K=B_{H}\left(H_{a}\right)$ and $X=\Delta_{\rho ,\sigma }$, which ensures that the inequality

(79) $$-\log\left(V_{s}^{†}\Delta_{\rho ,\sigma }V_{\rho }\right)\leq -V_{s}^{†}\log\left(\Delta_{\rho ,\sigma }\right)V_{\rho },$$

holds true, confirming the validity of the computations in (68), thereby establishing the monotonicity of the relative entropy.

### 3.2. Extension of Petz’s Proof to Non-Invertible Density Operators

The corrected version of Petz’s strategy for proving the monotonicity of relative entropy can be extended to include non-invertible density operators. To this end, the interplay between the support-based and the regularized definition of relative entropy given in (27) and (40), respectively, will prove to be useful.

First of all, note that, if supp(ρ)⊈supp(σ), then S(ρ∥σ)=+∞, and the monotonicity statement is trivially true.

We therefore restrict our attention to the case supp(ρ)⊆supp(σ), which ensures that the relative entropy is finite. Within this setting, we assume that ρ is non-invertible, while σ may or may not be invertible. This covers all cases not addressed by Petz’s original analysis in [10].

To establish the monotonicity of relative entropy in this broader context, we must first ensure that $S({\mathrm{Tr}}_{b}\left(\rho \right)∥{\mathrm{Tr}}_{b}\left(\sigma \right))<+\infty$, i.e., that $\mathrm{supp}\left({\mathrm{Tr}}_{b}\left(\rho \right)\right)\subseteq \mathrm{supp}\left({\mathrm{Tr}}_{b}\left(\sigma \right)\right)$. Observe that it is not necessary to check this condition when ρ,σ>0, since, in that case, ${\mathrm{Tr}}_{b}\left(\rho \right)$ and ${\mathrm{Tr}}_{b}\left(\sigma \right)$ are also positive definite, and, thus, their supports coincide with $H_{a}$.

We need a preliminary lemma regarding the kernel of positive semi-definite operators.

**Lemma** **1.** *Let $T_{1}$ and $T_{2}$ be Hermitian positive semi-definite operators on a finite-dimensional Hilbert space H. Then,*

(80) $$\ker\left(T_{1}+T_{2}\right)=\ker\left(T_{1}\right)\cap \ker\left(T_{2}\right).$$

**Proof.**  Proving the inclusion $\ker\left(T_{1}\right)\cap \ker\left(T_{2}\right)\subseteq \ker\left(T_{1}+T_{2}\right)$ is trivial: if we have $x\in \ker\left(T_{1}\right)\cap \ker\left(T_{2}\right)$, then $0_{H}=T_{1}x+T_{2}x=\left(T_{1}+T_{2}\right)x$, so $x\in \ker(T_{1}+T_{2})$.

To show the inclusion $\ker\left(T_{1}+T_{2}\right)\subseteq \ker\left(T_{1}\right)\cap \ker\left(T_{2}\right)$, we first note that, thanks to the positive semi-definiteness of $T_{1}$ and $T_{2}$, for all x∈H, we have

(81) $$\langle \left(T_{1}+T_{2}\right)x,x\rangle =\langle T_{1}x,x\rangle +\langle T_{2}x,x\rangle \geq 0,$$

with equality to 0 if and only if $\langle T_{1}x,x\rangle =\langle T_{2}x,x\rangle =0$, i.e., if and only if $T_{1}x=T_{2}x=0$ because the eigenvalues of both $T_{1}$ and $T_{2}$ are non-negative.

Hence, if $x\in \ker(T_{1}+T_{2})$, then $\langle \left(T_{1}+T_{2}\right)x,x\rangle =0$, so, due to the previous considerations, $T_{1}x=T_{2}x=0$, and, therefore, $x\in \ker\left(T_{1}\right)\cap \ker\left(T_{2}\right)$. □

**Proposition** **1.** *For all density operators ρ,σ such that supp(ρ)⊆supp(σ), it holds that*

(82) $$\mathrm{supp}\left({\mathrm{Tr}}_{b}\left(\rho \right)\right)\subseteq \mathrm{supp}\left({\mathrm{Tr}}_{b}\left(\sigma \right)\right).$$

**Proof.**  Using the notation of Section 2, consider again the spectral decompositions of ρ and σ:

(83) $$\rho =\sum_{j\in I_{+}\left(\rho \right)}\lambda_{j}P_{j}+0P_{0},\;\sigma =\sum_{k\in I_{+}\left(\sigma \right)}\mu_{k}\Pi_{k}+0\Pi_{0},$$

where $P_{j}$ and $\Pi_{k}$ are the orthogonal projectors onto the eigenspaces corresponding to the positive eigenvalues $\lambda_{j}$ of ρ and $\mu_{k}$ of σ, respectively. Then, the following operator sums yield the orthogonal projectors onto supp(ρ) and supp(σ), respectively:

(84) $$P=\sum_{j\in I_{+}\left(\rho \right)}P_{j},\;\Pi =\sum_{k\in I_{+}\left(\sigma \right)}\Pi_{k}.$$

Using the linearity and positivity of the partial trace and the fact that multiplying a positive semi-definite operator by a strictly positive scalar does not change its support, we have

(85) $$\begin{array}{cc} \mathrm{supp}\left({\mathrm{Tr}}_{b}\left(\rho \right)\right) & =\mathrm{supp}\left(\sum_{j\in I_{+}\left(\rho \right)}\lambda_{j}{\mathrm{Tr}}_{b}\left(P_{j}\right)\right)=\mathrm{supp}\left(\sum_{j\in I_{+}\left(\rho \right)}{\mathrm{Tr}}_{b}\left(P_{j}\right)\right)\\ & =\mathrm{supp}\left({\mathrm{Tr}}_{b}\left(\sum_{j\in I_{+}\left(\rho \right)}P_{j}\right)\right)=\mathrm{supp}\left({\mathrm{Tr}}_{b}\left(P\right)\right). \end{array}$$

The same argument applies to σ and Π so that

(86) $$\mathrm{supp}\left({\mathrm{Tr}}_{b}\left(\sigma \right)\right)=\mathrm{supp}\left({\mathrm{Tr}}_{b}\left(\Pi \right)\right).$$

Moreover, according to a standard result about orthogonal projectors, the inclusion supp(ρ)⊆supp(σ) is equivalent to the fact that the operator Q:=Π−P is an orthogonal projector on $\mathrm{supp}\left(\sigma \right)\cap \mathrm{supp}{\left(\rho \right)}^{⊥}=\mathrm{supp}\left(\sigma \right)\cap \ker\left(\rho \right)$. By the linearity of the partial trace, we can write

(87) $${\mathrm{Tr}}_{b}\left(\Pi \right)={\mathrm{Tr}}_{b}\left(P\right)+{\mathrm{Tr}}_{b}\left(Q\right),$$

and, moreover, using the fact that ${\mathrm{Tr}}_{b}$ is a positive map and Lemma 1, we have

(88) $$\begin{array}{cc} \mathrm{supp}({\mathrm{Tr}}_{b}\left(\Pi \right)) & =\mathrm{supp}({\mathrm{Tr}}_{b}\left(P\right)+{\mathrm{Tr}}_{b}\left(Q\right))\\ & ={\left[\ker({\mathrm{Tr}}_{b}\left(P\right)+{\mathrm{Tr}}_{b}\left(Q\right))\right]}^{⊥}\\ & ={\left[\ker\left({\mathrm{Tr}}_{b}\left(P\right)\right)\cap \ker\left({\mathrm{Tr}}_{b}\left(Q\right)\right)\right]}^{⊥}\\ & =\mathrm{span}(\ker{\left({\mathrm{Tr}}_{b}\left(P\right)\right)}^{⊥}\cup \ker{\left({\mathrm{Tr}}_{b}\left(Q\right)\right)}^{⊥})\\ & =\mathrm{supp}\left({\mathrm{Tr}}_{b}\left(P\right)\right)+\mathrm{supp}\left({\mathrm{Tr}}_{b}\left(Q\right)\right)⊇\mathrm{supp}\left({\mathrm{Tr}}_{b}\left(P\right)\right), \end{array}$$

having used a standard property of the orthogonal complement of the intersection of two vector subspaces. Therefore,

(89) $$\mathrm{supp}\left({\mathrm{Tr}}_{b}\left(\rho \right)\right)=\mathrm{supp}\left({\mathrm{Tr}}_{b}\left(P\right)\right)\subseteq \mathrm{supp}\left({\mathrm{Tr}}_{b}\left(\Pi \right)\right)=\mathrm{supp}\left({\mathrm{Tr}}_{b}\left(\sigma \right)\right),$$

as claimed. □

While the support-based definition of relative entropy is useful to prove Equation (82), in order to prove the monotonicity of relative entropy for a non-invertible density operator ρ, the regularized definition (40) turns out to be more adequate.

Since both $\rho_{\varepsilon }>0$ and $\sigma_{\varepsilon }>0$ for any arbitrarily small ε>0, the modular operator $\Delta_{\rho_{\varepsilon },\sigma_{\varepsilon }}$ is well-defined. Similarly, the modular operator $\Delta_{\rho_{\varepsilon },\sigma_{\varepsilon }}^{a}$ is also well-defined.

By applying the same steps used in the corrected version of Petz’s proof and using Equation (52), we obtain that, for any ε>0,

(90) $$\begin{array}{cc} \mathrm{Tr}[{\mathrm{Tr}}_{b}\left(\rho_{\varepsilon }\right)\log{\mathrm{Tr}}_{b}\left(\rho_{\varepsilon }\right)-{\mathrm{Tr}}_{b}\left(\rho_{\varepsilon }\right)\log{\mathrm{Tr}}_{b}\left(\sigma_{\varepsilon }\right)] & ={\langle {\mathrm{Tr}}_{b}{\left(\rho_{\varepsilon }\right)}^{1/2},-\log\left(\Delta_{\rho_{\varepsilon },\sigma_{\varepsilon }}^{a}\right){\mathrm{Tr}}_{b}{\left(\rho_{\varepsilon }\right)}^{1/2}\rangle }_{a}\\ & \leq {\langle \rho_{\varepsilon }^{1/2},-\log\left(\Delta_{\rho_{\varepsilon },\sigma_{\varepsilon }}\right)\rho_{\varepsilon }^{1/2}\rangle }_{ab}\\ & =\mathrm{Tr}[\rho_{\varepsilon }\log\rho_{\varepsilon }-\rho_{\varepsilon }\log\sigma_{\varepsilon }]. \end{array}$$

Notice that we can deal with $\rho_{\varepsilon },\sigma_{\varepsilon }$ instead of ρ,σ even if they do not have unit trace because this property is used in Petz’s original (and flawed) argument only in Equation (71), which does not play any role in the corrected version outlined in Section 3.1.

By continuity, taking the limit $\varepsilon \to 0^{+}$ on both sides of the previous inequality leads to

(91) $$\begin{array}{cc} S({\mathrm{Tr}}_{b}\left(\rho \right)||{\mathrm{Tr}}_{b}\left(\sigma \right)) & =\lim_{\epsilon \to 0^{+}}\mathrm{Tr}\left[{\mathrm{Tr}}_{b}\left(\rho_{\varepsilon }\right)\log{\mathrm{Tr}}_{b}\left(\rho_{\varepsilon }\right)-{\mathrm{Tr}}_{b}\left(\rho_{\varepsilon }\right)\log{\mathrm{Tr}}_{b}\left(\sigma_{\varepsilon }\right)\right]\\ & \;\leq \lim_{\epsilon \to 0^{+}}\mathrm{Tr}\left[\rho_{\varepsilon }\log\rho_{\varepsilon }-\rho_{\varepsilon }\log\sigma_{\varepsilon }\right]=S\left(\rho ||\sigma \right), \end{array}$$

which completes the proof for non-invertible ρ and general σ.

As a final remark, we note that, in [10], Petz claimed that his proof applies not only to quantum channels but also to adjoints of unital Schwarz maps. However, that claim relies on the flawed argument that we have pointed out. The validity of Petz’s proof can be restored by proving that $V_{\rho }$ is an isometry, a property that holds if we are dealing with the partial trace but not with the adjoint of a general unital Schwarz map.

## 4. Uhlmann’s Proof of the Monotonicity of the Relative Entropy Under Partial Trace

The proof offered by Uhlmann in [7] (see also [27] for a review) was more general than that offered by Petz because it also naturally encompassed the case of non-invertible density operators. However, thanks to the correction and generalization of Petz’s proof outlined in the previous section, now, we can state that the two procedures have the same generality.

Uhlmann’s proof is based on the concept of interpolations of positive sesquilinear forms, which we recall in the following subsection. Coherently with the analysis developed so far, we will consider only the case of finite dimensional vector spaces.

### 4.1. Interpolations of Positive Sesquilinear Forms

Let *V* be a vector space of finite dimension *d* over the field F=R or C, and let F(V) be the set of sesquilinear forms over *V*, assumed to be linear in the second variable and conjugate-linear in the first. The results of this subsection also encompass the case of *bilinear* forms.

We say that α∈F(V) is *positive* if α(v,v)≥0∀v∈V, and we denote the space of positive sesquilinear forms over *V* by $F_{+}\left(V\right)$.

We can endow $F_{+}\left(V\right)$ with a Löwner-like partial ordering: given $\alpha ,\beta \in F_{+}\left(V\right)$, we say that β≤α if α−β≥0.

Fixing a basis of *V*, for any form α∈F(V), there exists a unique Hermitian operator $T\in {\mathrm{End}}_{F}\left(V\right)$ such that, for all v,w∈V, written in coordinates as $x,y\in F^{d}$, one has

(92) $$\alpha \left(v,w\right)=x^{†}Ty.$$

It can be easily proven that, for a positive form $\alpha \in F_{+}\left(V\right)$, the kernel of α coincides with the *isotropic cone*

(93) ker(α)={v∈V:α(v,v)=0},

and they are both equal to the kernel of the positive semi-definite operator *T*.

Let H now be a Hilbert space with inner product ${\langle \;,\;\rangle }_{H}$, h:V→H be a linear surjective map, and $A\in B_{H}\left(H\right)$. Then, $\alpha \in F_{+}\left(V\right)$ is said to be *represented by*(H,h,A), indicated with α∼(H,h,A), if

(94) $$\alpha \left(v,w\right)={\langle h\left(v\right),A\;h\left(w\right)\rangle }_{H}\;,\;\forall v,w\in V.$$

Two representations of positive sesquilinear forms α∼(H,h,A) and β∼(H,h,B) are said to be *compatible* if [A,B]=0. As we shall see shortly, compatibility is a key concept in constructing a functional calculus for sesquilinear forms.

The following theorem shows that compatible representations exist, and its constructive proof provides a (non-unique) way to build them.

**Theorem** **2.** *Let $\alpha ,\beta \in F_{+}\left(V\right)$. Then, there exist representations α∼(H,h,A) and β∼(H,h,B) (with the same mapping h) such that [A,B]=0.*

**Proof.**  Let N⊂V be the kernel of the form α+β

(95) N={v∈V:α(v,v)+β(v,v)=0}.

By fixing a basis of *V*, we can associate α and β with two Hermitian and positive semi-definite operators $T_{1},T_{2}\in {\mathrm{End}}_{F}\left(V\right)$ via Equation (92). It follows that

(96) $$N=\ker\left(\alpha +\beta \right)=\ker\left(T_{1}+T_{2}\right)=\ker\left(T_{1}\right)\cap \ker\left(T_{2}\right),$$

where the last equality is provided by Lemma 1.

Now, by setting H:=V/N, we can define the following inner product:

(97) $$\begin{array}{cccc} \langle \;,\;\rangle : & H\times H & ⟶ & F\\ \; & \left(v+N,w+N\right) & ⟼ & \langle v+N,w+N\rangle =\alpha (v,w)+\beta (v,w). \end{array}$$

Note that this inner product is well-defined thanks to the equalities (96).

By taking *h* as the (surjective) quotient map h:V→H, v↦h(v)=v+N, we can define the following positive sesquilinear forms on H:

(98) $$\tilde{\alpha }\left(h\left(v\right),h\left(w\right)\right)=\tilde{\alpha }\left(v+N,w+N\right):=\alpha \left(v,w\right),$$

(99) $$\tilde{\beta }\left(h\left(v\right),h\left(w\right)\right)=\tilde{\beta }\left(v+N,w+N\right):=\beta \left(v,w\right),$$

for all v,w∈V.

Thanks to the Riesz representation theorem, there exists a unique couple of positive Hermitian operators $A,B\in B_{H}\left(H\right)$ such that

(100) $$\tilde{\alpha }\left(h\left(v\right),h\left(w\right)\right)=\langle h\left(v\right),A\;h\left(w\right)\rangle =\alpha \left(v,w\right),$$

(101) $$\tilde{\beta }\left(h\left(v\right),h\left(w\right)\right)=\langle h\left(v\right),B\;h\left(w\right)\rangle =\beta \left(v,w\right).$$

It follows that, for all v,w∈V,

(102) $$\begin{array}{cc} \langle h(v),h(w)\rangle & =\alpha \left(v,w\right)+\beta \left(v,w\right)=\tilde{\alpha }\left(h\left(v\right),h\left(w\right)\right)+\tilde{\beta }\left(h\left(v\right),h\left(w\right)\right)\\ & =\langle h(v),(A+B)\;h(w)\rangle , \end{array}$$

and, hence, $A+B=id_{H}$, which implies that *A* and *B* commute. □

Consider now $R_{+}^{2}=\left\{\left(x,y\right)\in R^{2}\;:\;x\geq 0,\;y\geq 0\right\}$, and let *J* be the set of homogeneous (of degree 1), measurable, and locally bounded functions $f:R_{+}^{2}\to R$. It is possible to develop the concept of function of positive sesquilinear forms thanks to the following theorem, whose quite lengthy proof can be consulted in [28].

**Theorem** **3.** *Let V be a vector space, $\alpha ,\beta \in F_{+}\left(V\right)$, and let α∼(H,h,A) and β∼(H,h,B) be two compatible representations of α and β. Then, for any f∈J, the function γ:V×V→F,*

(103) γ(v,w):=〈h(v),f(A,B)h(w)〉,v,w∈V,

*is a well-defined sesquilinear form on V; i.e., γ is independent of the choice of representations, and, for any given f, γ depends only on $\alpha ,\beta \in F_{+}\left(V\right)$.*

Note that, on the right-hand side of (103), f(A,B) is intended as an operator function, which is well-defined since *A* and *B* commute, and so they can be simultaneously diagonalized.

Combining Theorems 2 and 3, every f∈J can be extended to a function of positive sesquilinear forms. Keeping the same symbol for simplicity, we can define

(104) $$f:F_{+}\left(V\right)\times F_{+}\left(V\right)⟶F\left(V\right),$$

as follows: given $\alpha ,\beta \in F_{+}\left(V\right)$ and any compatible representations α∼(H,h,A) and β∼(H,h,B), the sequilinear form f(α,β) is represented as

(105) f(α,β)∼(H,h,f(A,B)).

The elements of *J* used to define the concept of the interpolation of positive sesquilinear forms are the positive functions $f^{t}\left(x,y\right)=x^{1-t}y^{t}$, where t∈[0,1]. Given $\alpha ,\beta \in F_{+}\left(V\right)$, we call *interpolation from α to β* the positive sesquilinear form

(106) $$\begin{array}{cccc} \gamma_{\alpha \to \beta }^{t}:=f^{t}\left(\alpha ,\beta \right): & V\times V & ⟶ & F\\ \; & \left(v,w\right) & ⟼ & f^{t}\left(\alpha ,\beta \right)\left(v,w\right), \end{array}$$

which means that, for any compatible representation α∼(H,h,A) and β∼(H,h,B),

(107) $$\begin{array}{cc} \gamma_{\alpha \to \beta }^{t}\left(v,w\right) & =\langle h\left(v\right),A^{1-t}\;B^{t}\;h\left(w\right)\rangle ,\;\forall v,w\in V. \end{array}$$

The interpolation is said to go from α to β because, clearly, $\gamma_{\alpha \to \beta }^{0}=\alpha$ and $\gamma_{\alpha \to \beta }^{1}=\beta$. Moreover, the interpolation of two interpolations is another interpolation. In fact, given $t_{1},t_{2}\in \left[0,1\right]$, we have

(108) $$\begin{array}{cc} \gamma_{\gamma_{\alpha \to \beta }^{t_{1}}\to \gamma_{\alpha \to \beta }^{t_{2}}}^{t}\left(v,w\right) & =\langle h\left(v\right),{\left(A^{1-t_{1}}B^{t_{1}}\right)}^{1-t}{\left(A^{1-t_{2}}B^{t_{2}}\right)}^{t}\;h\left(w\right)\rangle \\ & =\langle h\left(v\right),A^{1-t_{1}-t+t_{1}t}A^{t-t_{2}t}B^{t_{1}-t_{1}t}B^{t_{2}t}\;h\left(w\right)\rangle \\ & =\langle h\left(v\right),A^{1-(t_{1}\left(1-t\right)+t_{2}t)}B^{t_{1}\left(1-t\right)+t_{2}t}\;h\left(w\right)\rangle \\ & =\gamma_{\alpha \to \beta }^{t^{\prime }}\left(v,w\right), \end{array}$$

with $t^{\prime }=t_{1}\left(1-t\right)+t_{2}t$.

The interpolation of α,β computed at the value t=1/2 corresponds to a particularly important positive sesquilinear form, indicated with

(109) $$\sqrt{\alpha \beta }:=\gamma_{\alpha \to \beta }^{1/2},$$

and called the *geometric mean* of α,β. Clearly, we have

(110) $$\sqrt{\alpha \beta }\;\left(v,w\right)=\gamma_{\alpha \to \beta }^{1/2}\left(v,w\right)=\langle h\left(v\right),A^{1/2}B^{1/2}\;h\left(w\right)\rangle ,\;\forall v,w\in V.$$

An important property of the geometric mean of α and β will emerge in connection with the following concept: a positive sesquilinear form $r\in F_{+}\left(V\right)$ is said to be *dominated* by $\alpha ,\beta \in F_{+}\left(V\right)$ if

(111) $${\left|r\left(v,w\right)\right|}^{2}\leq \alpha \left(v,v\right)\beta \left(w,w\right),\;\forall v,w\in V.$$

The following theorem establishes that $\sqrt{\alpha \beta }$ is the ‘maximal’ positive sesquilinear form dominated by α and β.

**Theorem** **4.** *Let V be a vector space, and let $\alpha ,\beta \in F_{+}\left(V\right)$; then, $\sqrt{\alpha \beta }$ is dominated by α,β. Moreover, interpreting $F_{+}\left(V\right)$ as a partially ordered set, let $S\subset F_{+}\left(V\right)$ be the subset of positive sesquilinear forms dominated by α,β; then, $\sqrt{\alpha \beta }=\sup S$, i.e., $r\leq \sqrt{\alpha \beta }$ for all r∈S.*

**Proof.**  The first statement is simply an application of the Cauchy–Schwarz inequality. For all v,w∈V and for any compatible representations of α and β, we have

(112) $$\begin{array}{cc} |\langle h\left(v\right),A^{1/2}B^{1/2}h\left(w\right)\rangle {|}^{2} & =|\langle A^{1/2}h\left(v\right),B^{1/2}h\left(w\right)\rangle {|}^{2}\\ & \leq \langle A^{1/2}h\left(v\right),A^{1/2}h\left(v\right)\rangle \langle B^{1/2}h\left(w\right),B^{1/2}h\left(w\right)\rangle \\ & =\langle h(v),Ah(v)\rangle \langle h(w),Bh(w)\rangle , \end{array}$$

having used the fact that *A* and *B*, and so their square roots are Hermitian operators. Thus,

(113) $$|\sqrt{\alpha \beta }{\left(v,w\right)|}^{2}\leq \alpha \left(v,v\right)\beta \left(w,w\right),\;\forall v,w\in V,$$

so $\sqrt{\alpha \beta }$ is dominated by α and β.

To prove the second statement, consider a form $r\in F_{+}\left(V\right)$ dominated by α,β, and let α∼(H,h,A) and β∼(H,h,B) be compatible representations. By applying the constructive proof of Theorem 2 to *r*, we can find a positive operator $C\in B_{H}\left(H\right)$ such that r(v,w)=〈h(v),Ch(w)〉; however, the representation r∼(H,h,C) will not, in general, be compatible with that of α and β. Since *r* is dominated by α and β, we have

(114) $${\left|\langle h\left(v\right),Ch\left(w\right)\rangle \right|}^{2}\leq \langle h\left(v\right),Ah\left(v\right)\rangle \langle h\left(w\right),Bh\left(w\right)\rangle ,\;\forall v,w\in V,$$

or, thanks to the fact that *h* is surjective on H,

(115) $${\left|\langle x,Cy\rangle \right|}^{2}\leq \langle x,Ax\rangle \langle y,By\rangle ,\;\forall x,y\in H.$$

Now, the second statement of the theorem, i.e., $r\leq \sqrt{\alpha \beta }$, means that $\left(\sqrt{\alpha \beta }-r\right)\left(v,v\right)\geq 0$ for all v∈V and all r∈S, which is equivalent to $\langle u,\left(A^{1/2}B^{1/2}-C\right)u\rangle \geq 0$ for all u∈H and all *C* satisfying inequality (115).

It follows that the second statement of the theorem will be proven if we manage to show that $C\leq A^{1/2}B^{1/2}$. In order to obtain this result, a regularization procedure applied to the operators A,B will be helpful: since A≥0 and B≥0, for all ε>0, $A_{\varepsilon }:=A+\varepsilon id_{H}$, $B_{\varepsilon }:=B+\varepsilon id_{H}$, and their square roots $A_{\varepsilon }^{1/2}$, $B_{\varepsilon }^{1/2}$ are Hermitian, positive, and invertible. Moreover, it is clear that $A\leq A_{\epsilon }$, $B\leq B_{\epsilon }$, hence

(116) $$A_{\varepsilon }^{-1/2}AA_{\varepsilon }^{-1/2}\leq id_{H},\;B_{\varepsilon }^{-1/2}BB_{\varepsilon }^{-1/2}\leq id_{H}.$$

Finally, $A_{\varepsilon }$ and $B_{\varepsilon }$ are monotonically decreasing in ε w.r.t. the Löwner ordering and $A_{\varepsilon }\to A$ and $B_{\varepsilon }\to B$, as ε→0. For all x,y∈H, there exist unique vectors u,v∈H such that

(117) $$x:=A_{\varepsilon }^{-1/2}u,\;y:=B_{\varepsilon }^{-1/2}v,$$

then inequality (115) becomes

(118) $$|\langle A_{\varepsilon }^{-1/2}u,CB_{\varepsilon }^{-1/2}v\rangle {|}^{2}\leq \langle A_{\varepsilon }^{-1/2}u,AA_{\varepsilon }^{-1/2}u\rangle \langle B_{\varepsilon }^{-1/2}v,BB_{\varepsilon }^{-1/2}v\rangle ,$$

i.e.,

(119) $$|\langle u,A_{\varepsilon }^{-1/2}CB_{\varepsilon }^{-1/2}v\rangle {|}^{2}\leq \langle u,A_{\varepsilon }^{-1/2}AA_{\varepsilon }^{-1/2}u\rangle \langle v,B_{\varepsilon }^{-1/2}BB_{\varepsilon }^{1/2}v\rangle \leq \langle u,u\rangle \langle v,v\rangle ,$$

having used the inequalities written in (116).

By considering u=v and taking into account that $A_{\varepsilon }^{-1/2}CB_{\varepsilon }^{-1/2}$ is a positive operator, we can write

(120) $$\langle u,A_{\varepsilon }^{-1/2}CB_{\varepsilon }^{-1/2}u\rangle \leq \langle u,u\rangle ,\;\forall u\in H,$$

which implies that $A_{\epsilon }^{-1/2}CB_{\epsilon }^{-1/2}\leq id_{H}$, thus $C\leq A_{\varepsilon }^{1/2}B_{\varepsilon }^{1/2}$. By taking the limit ε→0, we get $C\leq A^{1/2}B^{1/2}$, and so the second statement of the theorem is also proven. □

The property of the geometric mean just proven allows us to extend the ordering relation between two positive sesquilinear forms to their interpolations, in the sense specified by the following theorem.

**Theorem** **5.** *Let V be a vector space, and let $\alpha ,\alpha^{\prime },\beta ,\beta^{\prime }\in F_{+}\left(V\right)$ such that $\alpha^{\prime }\leq \alpha$ and $\beta^{\prime }\leq \beta$; then,*

(121) $$\gamma_{\alpha^{\prime }\to \beta^{\prime }}^{t}\leq \gamma_{\alpha \to \beta }^{t},\;\forall t\in \left[0,1\right].$$

**Proof.**  The statement is clearly satisfied for t=0,1. Setting t=1/2, thanks to the first part of Theorem 4, we get

(122) $$|\gamma_{\alpha^{\prime }\to \beta^{\prime }}^{1/2}{\left(v,w\right)|}^{2}\leq \alpha^{\prime }\left(v,v\right)\beta^{\prime }\left(w,w\right)\leq \alpha \left(v,v\right)\beta \left(w,w\right),\;\forall v,w\in V,$$

where the second inequality follows from the hypotheses of this theorem. This means that $\gamma_{\alpha^{\prime }\to \beta^{\prime }}^{1/2}$ is dominated by α,β, and so the extremality of the geometric mean implies that

(123) $$\gamma_{\alpha^{\prime }\to \beta^{\prime }}^{1/2}\leq \gamma_{\alpha \to \beta }^{1/2}.$$

If we now use Equation (108) with $t=t_{2}=1/2$ and $t_{1}=0$, we get

(124) $$\gamma_{\alpha \to \beta }^{1/4}=\gamma_{\gamma_{\alpha \to \beta }^{0}\to \gamma_{\alpha \to \beta }^{1/2}}^{1/2},\;\gamma_{\alpha^{\prime }\to \beta^{\prime }}^{1/4}=\gamma_{\gamma_{\alpha^{\prime }\to \beta^{\prime }}^{0}\to \gamma_{\alpha^{\prime }\to \beta^{\prime }}^{1/2}}^{1/2}.$$

By repeating the previous argument, this time using Equation (123), we show that $\gamma_{\alpha^{\prime }\to \beta^{\prime }}^{1/4}\leq \gamma_{\alpha \to \beta }^{1/4}$.

By iterating this procedure, we can prove the statement of the theorem for any t∈[0,1] of the type $t_{k,n}=k/2^{n}$, with n,k∈N, $k\leq 2^{n}$, which is a dense subset of [0,1].

Finally, the functions $t_{k,n}\to \gamma_{\alpha \to \beta }^{t_{k,n}}\left(v,w\right)$ and $t_{k,n}\to \gamma_{\alpha^{\prime }\to \beta^{\prime }}^{t_{k,n}}\left(v,w\right)$ are continuous for every fixed v,w∈V; hence, the theorem holds for all t∈[0,1]. □

In a similar way, we can prove another important result. Let ψ:U→V be a linear map between vector spaces, and let $\alpha ,\beta \in F_{+}\left(V\right)$. Then, ψ allows us to *pull back* these sesquilinear forms on *U* as follows:

(125) $$\begin{array}{cccc} \psi^{*}\alpha : & U\times U & ⟶ & F\\ \; & \left(v,w\right) & ⟼ & \alpha (\psi (v),\psi (w)),\\ \; & \; & & \\ \psi^{*}\beta : & U\times U & ⟶ & F\\ \; & \left(v,w\right) & ⟼ & \beta (\psi (v),\psi (w)). \end{array}$$

The following theorem shows that the pull-back of an interpolation of the forms in $F_{+}\left(V\right)$ is always ‘smaller’ than the interpolation of their pull-backs, with respect to the partial ordering of $F_{+}\left(U\right)$.

**Theorem** **6.** *Let U,V be vector spaces, ψ:U→V be a linear map, and $\alpha ,\beta \in F_{+}\left(V\right)$; then,*

(126) $$\psi^{*}\gamma_{\alpha \to \beta }^{t}\leq \gamma_{\psi^{*}\alpha \to \psi^{*}\beta }^{t},\;\forall t\in \left[0,1\right].$$

**Proof.**  The argument that we use is quite similar to the one appearing in the previous proof. The statement is true for t=0 and t=1 because, in these cases, we have $\psi^{*}\alpha \leq \psi^{*}\alpha$ and $\psi^{*}\beta \leq \psi^{*}\beta$, respectively.

Let us now consider t=1/2, which gives rise to the geometric mean, so

(127) $$|\psi^{*}\sqrt{\alpha \beta }{\left(v,w\right)|}^{2}={\left|\sqrt{\alpha \beta }\left(\psi \left(v\right),\psi \left(w\right)\right)\right|}^{2},\;\forall v,w\in U.$$

Since $\sqrt{\alpha \beta }$ is dominated by α,β, we can write

(128) $$|\psi^{*}\sqrt{\alpha \beta }{\left(v,w\right)|}^{2}\leq \alpha \left(\psi \left(v\right)\psi \left(v\right)\right)\beta \left(\psi \left(w\right),\psi \left(w\right)\right)=\psi^{*}\alpha \left(v,v\right)\psi^{*}\beta \left(w,w\right),$$

which shows that $\psi^{*}\sqrt{\alpha \beta }$ is dominated by $\psi^{*}\alpha$, $\psi^{*}\beta$. Thus, by the extremal property of the geometric mean, the statement of the theorem holds for t=1/2.

By iterating this reasoning as done in the proof of the previous theorem, the validity of inequality (126) can be generalized to all t∈[0,1]. □

### 4.2. Definition of the Relative Entropy in Terms of Interpolations of Forms

In this subsection, we apply the results previously established to reformulate the relative entropy in a manner that will facilitate the proof of its monotonicity under partial trace, which is to be presented in the next subsection.

Adopting notations analogous to those introduced at the beginning of Section 3, we identify the vector space *V*, on which the forms of interest for us will be defined, with $B(H_{ab})$. As we know, this is a Hilbert space w.r.t. the Hilbert–Schmidt inner product ${\langle A,B\rangle }_{ab}=\mathrm{Tr}\left(A^{†}B\right)$, $A,B\in B(H_{ab})$, which is a positive definite sesquilinear form.

Given two density operators $\rho ,\sigma \in B_{H}\left(H_{ab}\right)$, we can define the following positive sesquilinear forms $\rho_{L},\sigma_{R}:B\left(H_{ab}\right)\times B\left(H_{ab}\right)\to F$:

(129) $$\rho_{L}\left(A,B\right):=\mathrm{Tr}\left(\rho BA^{†}\right)=\mathrm{Tr}\left(A^{†}\rho B\right)=\langle A,L_{\rho }B\rangle ,$$

(130) $$\sigma_{R}\left(A,B\right):=\mathrm{Tr}\left(\sigma A^{†}B\right)=\mathrm{Tr}\left(A^{†}B\sigma \right)=\langle A,R_{\sigma }B\rangle ,$$

where the operators $L_{\rho }$ and $R_{\sigma }$ are defined as in Equation (46). We immediately recognize the two representations

(131) $$\rho_{L}∼\left(B\left(H_{ab}\right),id_{ab},L_{\rho }\right),\;\sigma_{R}∼\left(B\left(H_{ab}\right),id_{ab},R_{\sigma }\right),$$

where $id_{ab}$ is the identity map on $B(H_{ab})$. These representations are compatible because, thanks to Equation (47), $[L_{\rho },R_{\sigma }]=0$.

We can now define the *relative entropy positive sesquilinear form* between the two density operators ρ,σ, indicated with $S_{\rho ∥\sigma }:B\left(H_{ab}\right)\times B\left(H_{ab}\right)\to F$, as the rate of change of the interpolation $\gamma_{\rho_{L}\to \sigma_{R}}^{t}$ with respect to $\rho_{L}$

(132) $$S_{\rho ∥\sigma }\left(A,B\right)=-\underset{t\to 0^{+}}{lim inf}\frac{\gamma_{\rho_{L}\to \sigma_{R}}^{t}\left(A,B\right)-\rho_{L}\left(A,B\right)}{t},\;A,B\in B\left(H_{ab}\right).$$

**Remark** **1.** *The use of lim inf instead of an ordinary limit is motivated by the following two arguments:*

*1.*  *When ρ and σ are not invertible, the interpolation function $t\mapsto \gamma_{\rho_{L}\to \sigma_{R}}^{t}\left(A,B\right)$ may not be differentiable at $t=0^{+}$. The ordinary limit of the differential quotient at $t=0^{+}$ might fail to exist due to oscillations or a lack of smoothness in the interpolation path. In such situations, the ordinary limit does not exist. However, lim inf always exists (possibly infinite), thereby ensuring that the entropy form $S_{\rho ∥\sigma }\left(A,B\right)$ is always well-defined.*
*2.*  *The function $t\mapsto \gamma_{\rho_{L}\to \sigma_{R}}^{t}\left(A,B\right)$ is convex in t, as it arises from the interpolation $f_{t}\left(x,y\right)=x^{1-t}y^{t},$ which is jointly operator convex for x,y>0. For convex functions, the left and right derivatives at an endpoint may differ, and the correct notion of derivative from the right at t=0 is the lower right Dini derivative, i.e., the* lim inf *of the difference quotient. Thus, the use of* lim inf *aligns with standard practice in convex analysis.*

*The relative entropy between states ρ,σ can be recovered as follows.*

**Theorem** **7.** *For all density operators $\rho ,\sigma \in B_{H}\left(H_{ab}\right)$, we have*

(133) $$S\left(\rho ∥\sigma \right)=S_{\rho ∥\sigma }\left(id_{ab},id_{ab}\right).$$

**Proof.**  Let us first consider the case of invertible density operators ρ>0 and σ>0. Then,

(134) $$\begin{array}{cc} S_{\rho ∥\sigma }\left(id_{ab},id_{ab}\right) & =-\underset{t\to 0^{+}}{lim inf}\frac{\gamma_{\rho_{L}\to \sigma_{R}}^{t}\left(id_{ab},id_{ab}\right)-\rho_{L}\left(id_{ab},id_{ab}\right)}{t}\\ & =-\underset{t\to 0^{+}}{lim inf}\frac{\langle id_{ab},L_{\rho }^{1-t}R_{\sigma }^{t}id_{ab}\rangle -\langle id_{ab},L_{\rho }id_{ab}\rangle }{t}\\ & =-\underset{t\to 0^{+}}{lim inf}\frac{\mathrm{Tr}\left(\rho^{1-t}\sigma^{t}\right)-\mathrm{Tr}\left(\rho \right)}{t}=-{\left.\frac{d}{dt}\right|}_{t=0}\mathrm{Tr}(\rho^{1-t}\sigma^{t})\\ & =-{\left.\frac{d}{dt}\right|}_{t=0}\mathrm{Tr}\left(\exp((1-t)\log\rho )\exp(t\log\sigma )\right)\\ & =-\mathrm{Tr}[-\exp((1-t)\log\rho )\log\rho \exp(t\log\sigma )\\ & \;\;\;\;+\exp\left(\left(1-t\right)\log\rho \right)\exp\left(t\log\sigma \right)\log\sigma {]}_{t=0}\\ & =-\mathrm{Tr}\left(-\rho \log\rho +\rho \log\sigma \right)=\mathrm{Tr}\left(\rho \log\rho -\rho \log\sigma \right)\\ & =S(\rho ∥\sigma ). \end{array}$$

In the case of σ and ρ, which are not invertible, we can use the regularized version of relative entropy. We have seen an equivalent definition of the relative entropy in (40), and, thus, it is natural to consider

(135) $$\lim_{\epsilon \to 0^{+}}S_{\rho_{\epsilon }∥\sigma_{\epsilon }}\left(id_{ab},id_{ab}\right).$$

Now, we have that both $\rho_{\epsilon },\sigma_{\epsilon }$ are positive definite, and, thus, $\log\rho_{\epsilon }$, $\log\sigma_{\epsilon }$ are well-defined. By repeating the steps in (134), we get that

(136) $$\lim_{\epsilon \to 0^{+}}S_{\rho_{\epsilon }∥\sigma_{\epsilon }}\left(id_{ab},id_{ab}\right)=\lim_{\epsilon \to 0^{+}}\mathrm{Tr}\left(\rho_{\epsilon }\log\rho_{\epsilon }-\rho_{\epsilon }\log\sigma_{\epsilon }\right).$$

We obtain the same definition of relative entropy as in (27). □

### 4.3. Proof of the Monotonicity of the Relative Entropy Under Partial Trace

Thanks to formula (133), the monotonicity of the relative entropy under the partial trace ${\mathrm{Tr}}_{b}:B\left(H_{ab}\right)\to B\left(H_{a}\right)$ will be proven if we show that

(137) $$S_{{\mathrm{Tr}}_{b}\left(\rho \right)∥{\mathrm{Tr}}_{b}\left(\sigma \right)}\left(id_{a},id_{a}\right)\leq S_{\rho ∥\sigma }\left(id_{ab},id_{ab}\right).$$

As in Petz’s proof, the adjoint of the partial trace plays a central, though conceptually distinct, role in establishing the monotonicity of relative entropy.

Specifically, in Uhlmann’s approach, ${\mathrm{Tr}}_{b}^{†}$ acts as a pull-back map; i.e., we define $\psi :={\mathrm{Tr}}_{b}^{†}:B\left(H_{a}\right)\to B\left(H_{ab}\right)$ and use it to pull back the positive sesquilinear forms $\rho_{L}$ and $\sigma_{R}$ introduced in Equations (129) and (130), respectively. Thanks to Theorem 6, we have

(138) $$\psi^{*}\gamma_{\rho_{L}\to \sigma_{R}}^{t}\leq \gamma_{\psi^{*}\rho_{L}\to \psi^{*}\sigma_{R}}^{t},$$

i.e.,

(139) $$\gamma_{\rho_{L}\to \sigma_{R}}^{t}\left({\mathrm{Tr}}_{b}^{†}\left(X\right),{\mathrm{Tr}}_{b}^{†}\left(X\right)\right)=\psi^{*}\gamma_{\rho_{L}\to \sigma_{R}}^{t}\left(X,X\right)\leq \gamma_{\psi^{*}\rho_{L}\to \psi^{*}\sigma_{R}}^{t}\left(X,X\right),$$

for all $X\in B(H_{a})$. Now, we can use the fact that ${\mathrm{Tr}}_{b}$ preserves density operators and that ${\mathrm{Tr}}_{b}^{†}$ is a Schwarz map to write

(140) $$\begin{array}{cc} \psi^{*}\rho_{L}\left(X,X\right) & =\rho_{L}\left({\mathrm{Tr}}_{b}^{†}\left(X\right),{\mathrm{Tr}}_{b}^{†}\left(X\right)\right)=\mathrm{Tr}\left({\mathrm{Tr}}_{b}^{†}{\left(X\right)}^{†}\rho {\mathrm{Tr}}_{b}^{†}\left(X\right)\right)=\mathrm{Tr}\left(\rho {\mathrm{Tr}}_{b}^{†}\left(X\right){\mathrm{Tr}}_{b}^{†}{\left(X\right)}^{†}\right)\\ & \leq \mathrm{Tr}\left(\rho {\mathrm{Tr}}_{b}^{†}\left(XX^{†}\right)\right)=\langle \rho ,{\mathrm{Tr}}_{b}^{†}\left(XX^{†}\right)\rangle =\langle {\mathrm{Tr}}_{b}\left(\rho \right),XX^{†}\rangle =\mathrm{Tr}\left({\mathrm{Tr}}_{b}\left(\rho \right)XX^{†}\right)\\ & ={\mathrm{Tr}}_{b}{\left(\rho \right)}_{L}\left(X,X\right). \end{array}$$

Replacing $\rho_{L}$ with $\sigma_{R}$, we find

(141) $$\psi^{*}\sigma_{R}\left(X,X\right)\leq {\mathrm{Tr}}_{b}{\left(\sigma \right)}_{R}\left(X,X\right).$$

Thus, we have proven that $\psi^{*}\rho_{L}\leq {\mathrm{Tr}}_{b}{\left(\rho \right)}_{L}$ and $\psi^{*}\sigma_{R}\leq {\mathrm{Tr}}_{b}{\left(\sigma \right)}_{R}$, and so Theorem 5 implies that, for all t∈[0,1], it holds that

(142) $$\gamma_{\psi^{*}\rho_{L}\to \psi^{*}\sigma_{R}}^{t}\leq \gamma_{{\mathrm{Tr}}_{b}{\left(\rho \right)}_{L}\to {\mathrm{Tr}}_{b}{\left(\sigma \right)}_{R}}^{t}.$$

This result and inequality (139) imply

(143) $$\gamma_{\rho_{L}\to \sigma_{R}}^{t}\left({\mathrm{Tr}}_{b}^{†}\left(X\right),{\mathrm{Tr}}_{b}^{†}\left(X\right)\right)\leq \gamma_{{\mathrm{Tr}}_{b}{\left(\rho \right)}_{L}\to {\mathrm{Tr}}_{b}{\left(\sigma \right)}_{R}}^{t}\left(X,X\right),$$

for all $X\in B(H_{a})$. By considering in particular $X=id_{a}$ and recalling that ${\mathrm{Tr}}_{b}^{†}$ is a unital map, i.e., ${\mathrm{Tr}}_{b}^{†}\left(id_{a}\right)=id_{ab}$, we obtain

(144) $$\begin{array}{c} \gamma_{\rho_{L}\to \sigma_{R}}^{t}\left(id_{ab},id_{ab}\right)\leq \gamma_{{\mathrm{Tr}}_{b}{\left(\rho \right)}_{L}\to {\mathrm{Tr}}_{b}{\left(\sigma \right)}_{R}}^{t}\left(id_{a},id_{a}\right). \end{array}$$

Now, since ${\mathrm{Tr}}_{b}$ is trace-preserving, we have

(145) $$\begin{array}{cc} {\mathrm{Tr}}_{b}{\left(\rho \right)}_{L}\left(id_{a},id_{a}\right) & ={\langle id_{a},L_{{\mathrm{Tr}}_{b}\left(\rho \right)}id_{a}\rangle }_{a}=\mathrm{Tr}\left({\mathrm{Tr}}_{b}\left(\rho \right)\right)=\mathrm{Tr}\left(\rho \right)={\langle id_{ab},L_{\rho }id_{ab}\rangle }_{ab}\\ & =\rho_{L}\left(id_{ab},id_{ab}\right), \end{array}$$

and, thus, we can rewrite inequality (144) as follows:

(146) $$\gamma_{\rho_{L}\to \sigma_{R}}^{t}\left(id_{ab},id_{ab}\right)-\rho_{L}\left(id_{ab},id_{ab}\right)\leq \gamma_{{\mathrm{Tr}}_{b}{\left(\rho \right)}_{L}\to {\mathrm{Tr}}_{b}{\left(\sigma \right)}_{R}}^{t}\left(id_{a},id_{a}\right)-{\mathrm{Tr}}_{b}{\left(\rho \right)}_{L}\left(id_{a},id_{a}\right).$$

Thanks to Equations (133) and (132), this last inequality implies the monotonicity of the relative entropy under partial trace.

## 5. Conclusions

We revisited the monotonicity of relative entropy under the action of quantum channels by focusing on two important proofs: those by Petz and Uhlmann. While both approaches are foundational, their complexity has often hindered their pedagogical dissemination.

Our aim was to clarify and reconstruct these strategies within a finite-dimensional operator framework. In particular, we pointed out a subtle flaw in Petz’s original argument, whose validity was nonetheless restored by Petz and Nielsen soon after, and we showed how to rigorously extend this approach to incorporate non-invertible density operators.

It is also worth noting that our explicit construction of the isometric operator defined in Equation (60) sheds new light on its structural role within the broader context of quantum information theory. In particular, this operator can be seen as an essential component of the *Petz recovery map*, originally introduced in [9] in the setting of von Neumann algebras. Given a quantum channel C and a fixed full-rank state $\sigma \in B_{H}\left(H_{ab}\right)$, the Petz recovery map associated with C and σ is defined by

(147) $$P_{\sigma ,C}\left(\rho \right):=\sigma^{1/2}\;C^{†}\left(C{\left(\sigma \right)}^{-1/2}\;\rho \;C{\left(\sigma \right)}^{-1/2}\right)\;\sigma^{1/2},$$

or, equivalently, in terms of superoperators,

(148) $$P_{\sigma ,C}=L_{\sigma^{1/2}}\circ R_{\sigma^{1/2}}\circ C^{†}\circ R_{C{\left(\sigma \right)}^{-1/2}}^{a}\circ L_{C{\left(\sigma \right)}^{-1/2}}^{a}.$$

When $C={\mathrm{Tr}}_{b}$, this reduces to

(149) $$\begin{array}{cc} P_{\sigma ,{\mathrm{Tr}}_{b}} & =L_{\sigma^{1/2}}\circ \left(R_{\sigma^{1/2}}\circ {\mathrm{Tr}}_{b}^{†}\circ R_{{\mathrm{Tr}}_{b}{\left(\sigma \right)}^{-1/2}}^{a}\right)\circ L_{{\mathrm{Tr}}_{b}{\left(\sigma \right)}^{-1/2}}^{a}\\ & =L_{\sigma^{1/2}}\circ V_{\sigma }\circ L_{{\mathrm{Tr}}_{b}{\left(\sigma \right)}^{-1/2}}^{a}, \end{array}$$

or

(150) $$V_{\sigma }=L_{\sigma^{-1/2}}\circ P_{\sigma ,{\mathrm{Tr}}_{b}}\circ L_{{\mathrm{Tr}}_{b}{\left(\sigma \right)}^{1/2}}^{a}.$$

So, just as the Petz recovery map characterizes the reversibility of quantum channels and identifies conditions for saturation of the monotonicity inequality, the operator $V_{\sigma }$ explicitly captures the mechanism by which relative entropy is contracted under partial trace.

In recent developments, particularly in the work of Fawzi and Renner [29], the Petz recovery map plays a central role in quantitative refinements of the data processing inequality. Specifically, for states ρ and σ and a channel C, the inequality

(151) $$S\left(\rho ∥\sigma \right)-S\left(C\left(\rho \right)∥C\left(\sigma \right)\right)\geq -2\log F\left(\rho ,\left(P_{\sigma ,C}\circ C\right)\left(\rho \right)\right),$$

bounds the loss of distinguishability in terms of the fidelity *F* between the original state ρ and its recovered approximation via $P_{\sigma ,C}\circ C$.

The explicit identification of $V_{\sigma }$ offers a concrete realization of this recovery mechanism, reinforcing its interpretive clarity and suggesting further applications in entropy inequalities and recoverability conditions.

## Data Availability

The original contributions presented in this study are included in the article. Further inquiries can be directed to the corresponding author.
